# Oscillatory signatures underlie growth regimes in Arabidopsis pollen tubes: computational methods to estimate tip location, periodicity, and synchronization in growing cells

**DOI:** 10.1093/jxb/erx032

**Published:** 2017-03-28

**Authors:** Daniel S C Damineli, Maria Teresa Portes, José A Feijó

**Affiliations:** Department of Cell Biology and Molecular Genetics, University of Maryland, College Park, MD, USA

**Keywords:** Apical growth, biological oscillations, calcium, ion dynamics, kymograph, pollen tubes, spikes, synchronization, ultradian rhythms, wavelets

## Abstract

Oscillations in pollen tubes have been reported for many cellular processes, including growth, extracellular ion fluxes, and cytosolic ion concentrations. However, there is a shortage of quantitative methods to measure and characterize the different dynamic regimes observed. Herein, a suite of open-source computational methods and original algorithms were integrated into an automated analysis pipeline that we employed to characterize specific oscillatory signatures in pollen tubes of *Arabidopsis thaliana* (Col-0). Importantly, it enabled us to detect and quantify a Ca^2+^ spiking behaviour upon growth arrest and synchronized oscillations involving growth, extracellular H^+^ fluxes, and cytosolic Ca^2+^, providing the basis for novel hypotheses. Our computational approach includes a new tip detection method with subpixel resolution using linear regression, showing improved ability to detect oscillations when compared to currently available methods. We named this data analysis pipeline ‘Computational Heuristics for Understanding Kymographs and aNalysis of Oscillations Relying on Regression and Improved Statistics’, or CHUKNORRIS. It can integrate diverse data types (imaging, electrophysiology), extract quantitative and time-explicit estimates of oscillatory characteristics from isolated time series (period and amplitude) or pairs (phase relationships and delays), and evaluate their synchronization state. Here, its performance is tested with ratiometric and single channel kymographs, ion flux data, and growth rate analysis.

## Teaser

A data analysis pipeline that integrates diverse data sources, detects tip growth in kymographs, and analyses oscillations and synchronization reveales oscillatory signatures underlying distinct growth regimes in Arabidopsis pollen tubes.

## Introduction

Pollen tubes (PTs) are highly polarized cells that employ temporally and spatially coordinated cellular processes to achieve apical growth rates that are among the fastest known in nature (Boavida *et al.*, 2005). Oscillatory behaviour in PTs (reviewed in Feijó *et al.*, 2001; Portes *et al.*, 2015; Damineli *et al.*, 2017*b*) may occur within a characteristic range of frequencies found in apical growth and related cellular processes, such as tip-localized ion fluxes (Feijó *et al.*, 2001), intracellular ion concentration (Ca^2+^, H^+^, Cl^−^; Holdaway-Clarke *et al.*, 1997), cytoskeleton dynamics (F-actin; Fu *et al.*, 2001), membrane trafficking (Parton *et al.*, 2001), ROP signalling (Hwang *et al.*, 2005), NAD(P)H levels (Rounds *et al.*, 2010), and cell wall synthesis (Pierson *et al.*,1995; Rounds *et al.*, 2014). However the identity of the ‘pacemaker(s)’ underlying the oscillations and their physiological role in growth, cell polarity, and guidance is yet to be determined (Damineli *et al.*, 2017*b*). Furthermore, the quantification of the frequency/period, amplitude, and phase relationships of these oscillations is still limited by heterogeneity in spatial/temporal resolution and noise inherent to acquisition and analysis methods.

*Arabidopsis thaliana* is a prime system to investigate the molecular players involved in PT growth and fertilization, in which the wide array of available genetic tools calls for efforts to improve the resolution of functional analyses of ion transporters and other membrane-based mechanisms (Michard *et al.*, 2017). Yet the reduced dimensions of PTs pose experimental challenges given their diameter of approximately 5 μm, that is 2–3-fold smaller than other model species like tobacco (*Nicotiana tabacum*) or lily (*Lilium longiflorum*) (Holdaway-Clarke *et al.*, 1997; Gutermuth *et al.*, 2013). Although oscillations in Arabidopsis PTs grown *in vitro* have not been properly characterized, cytosolic Ca^2+^ ([Ca^2+^]_cyt_) oscillations were reported in male–female interaction preceding and during fertilization (Iwano *et al.*, 2012; Denninger *et al.*, 2014; Hamamura *et al.*, 2014; Ngo *et al.*, 2014). In addition, quantitative analysis of oscillatory behaviour was pivotal for the identification of subtle phenotypes in Glutamate Receptor-Like channel (GLR) 1.2 mutants (Michard *et al.*, 2011). On the other hand, the lack of proper quantification of [Ca^2+^]*cyt* oscillatory behaviour in mutants of a cyclic nucleotide-gated channel, *cngc18*, led to questionable extrapolations about its role on PT growth *in vitro* (Gao *et al.*, 2015). Thus, a precise phenotypic characterization of PTs grown *in vitro* or in the context of fertilization involving these oscillations would greatly benefit from adequate spatiotemporal resolution in data acquisition and statistical approaches.

Biological oscillations can be complex as they may have time-varying components, such as changes in baseline, frequency/period, amplitude, or waveform. These changes can reflect crucial transitions between different regulatory regimes. For example, the synchronization between different processes is of particular interest and has been implicated in polarity establishment, cell growth, and movement in general (Huang *et al.*, 2013; Wu and Lew, 2013; McClure and Lew, 2014). A synchronized state is characterized by a constant phase relationship between distinct oscillatory processes, which may only occur transiently or under specific circumstances (Wu *et al.*, 2013; Hoeller *et al.*, 2016; Lancaster *et al.*, 2016). However, typical analyses often do not capture the time-varying nature of these transitions and can provide inaccurate estimates of parameters of interest. For example, commonly used methods assume that oscillations have constant properties throughout time, providing a single estimate of significant periods (as with Fourier and auto-correlation analyses) or phase relationships/delays (as with cross-correlation analysis) for all time points (Holdaway-Clarke *et al.*, 1997; Messerli *et al.*, 1999; Rojas *et al.*, 2011). Together with the existence of noise either generated intrinsically in the cell or originating from the measurements, analysing oscillations and synchronization in such processes within a single cell calls for methods with greater spatial and temporal precision than the ones mostly employed in the PT and plant reproduction field.

Regarding the study of apical growth, rough estimates of the PT tip location are extracted from kymographs (a plot of single dimension of space through time) or with dedicated software applied to videos (Certal *et al.*, 2008; Gutermuth *et al.*, 2013; Portes *et al.* 2015). While there has been a wide array of methods used to detect the PT tip and track growth or changes in tip morphology, some of which achieve resolution below the pixel limit (subpixel), all methods developed so far involve either complex algorithms or model fitting (Holdaway-Clarke *et al.*, 1997; Messerli *et al.*, 1999; Rojas *et al.*, 2011; Haduch-Sendecka *et al.*, 2014; Tambo *et al.*, 2015*a*, *b*; Tambo and Bhanu, 2016). Furthermore, despite the availability of methods for cell tracking in general (Masuzzo *et al.*, 2016), to the best of our knowledge, a direct evaluation of their ability to detect oscillations in growth is lacking. Finally, the distinct data types involved in assaying multiple oscillations (e.g. imaging with electrophysiology) with different formats, resolution, and contaminations pose problems for understanding cell migration in general, creating the demand for tools that integrate distinct data types and yield comparable analyses (Masuzzo *et al.*, 2016). Given the diversity of methodology involved, a data analysis pipeline based on open-source programming language could provide an essential tool under such a scenario, since it enables full access, understanding, modification, and extension of the algorithms and workflow.

Here, we present a suite of computational methods based on the open-source programming language R to tackle oscillations at the cellular level. It introduces a precise algorithm to detect tip growth and oscillations applied to kymographs, providing subpixel resolution with a model-free approach that, although based on linear regression, it does not resort to assumptions about tip morphology. This method allows novel techniques to analyse single channel and ratiometric kymographs, which are available in distinct modules. The suite includes an automated pipeline that can take any time series composed of a time vector and a variable, reduce noise, outliers and artefacts, and analyse its time-varying period, phase, and amplitude. It can also evaluate the synchronization between two arbitrary time series by extracting their joint periodicity, phase relationship, and delays as a function of time. In summary, we introduce a powerful heuristic method to detect tip growth in kymographs with improved statistical methods to analyse oscillations, which we named ‘*C*omputational *H*euristics for *U*nderstanding *K*ymographs and a*N*alysis of *O*scillations *R*elying on *R*egression and *I*mproved *S*tatistics’ or CHUKNORRIS.

## Materials and methods

**Fig. 1.**
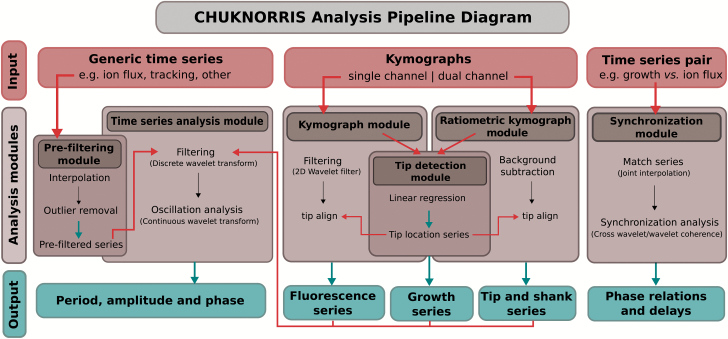
Diagram of the data analysis pipeline. The method we propose is a Computational Heuristics for Understanding Kymographs and aNalysis of Oscillations Relying on Regression and Improved Statistics (CHUKNORRIS), comprising five modules. Inputs to the modules are shown in red arrows while outputs are shown in blue (boxes and arrows). The pipeline was designed to analyse multiple data types, including generic time series, individually or in pairs, single channel, and ratiometric kymographs. Each analysis module has its corresponding R script and a basic usage tutorial available at https://github.com/damineli/CHUKNORRIS (last accessed 15 February 2017).

The main analysis pipeline is presented in Fig. 1 with scripts, data, and supporting material, including a basic usage tutorial, provided in Supplementary Data S1 (available at *JXB* online) and in the online repository GitHub (https://github.com/damineli/CHUKNORRIS, last accessed 15 February 2017). Raw data is also available in the online repository Dryad (Damineli *et al.*, 2017*a*).

### Analysis packages and details

All analyses and plots were performed with the statistical programming language R (R Core Team, 2016). Besides the original algorithms and code provided, specific pre-written packages serve as pillars of the pipeline: ‘wavelets’ for discrete wavelet analysis (Aldrich, 2013); ‘biwavelet’ for continuous wavelet, cross wavelet and wavelet coherence transforms (Gouhier *et al.*, 2016); ‘waveslim’ for 2D wavelet transform (Whitcher, 2016); and ‘tsoutliers’ for outlier removal in time series (López-de-Lacalle, 2016). Estimates of mean and standard deviation of distributions of period and delay were performed with a two-component Gaussian mixture model using the package ‘mixtools’ (Benaglia *et al.*, 2009).

### Data sources

#### Extracellular proton flux measurements

The ion-selective vibrating probe (Kühtreiber and Jaffe, 1990; Shipley and Feijó, 1999) was used to estimate extracellular H^+^ flux at the tip of PTs. Arabidopsis pollen grains were collected from fresh flowers and then germinated in liquid medium containing 500 μM KCl, 500 μM CaCl_2_, 125 μM MgSO_4_, 0.005% H_3_BO_3_, 125 μM HEPES, and 16% sucrose at pH 7.5. Pollen grains were incubated at 21.5°C for at least 3 h. Growing PTs longer than 150 µm were assayed using previously described protocols for measuring H^+^-specific fluxes (Certal *et al.*, 2008). Data acquisition, voltage parameters, and control of the 3D electrode micromanipulator were performed with the ASET software (Science Wares and Applicable Electronics). Simultaneous widefield-imaging of Arabidopsis PTs were performed on a custom-made rig using an inverted Nikon Eclipse TE300 equipped with an Andor iXon3 camera on the Koheler bottom port, and a Lumen 200Pro Fluorescence Illumination System. Yellow fluorescent protein (YFP) signal (excitation 498/510 nm, emission 535/559 nm) was acquired simultaneously to H^+^ flux measurements.

#### Ratiometric [Ca^2+^]_cyt_ imaging

Transgenic Arabidopsis PTs (Col-0) expressing the ratiometric Ca^2+^ probe YC3.6 (Nagai *et al.*, 2004) under the LAT52 promoter (Twell *et al.*, 1991) were imaged every 4 s on an Applied Precision Deltavision Core system, mounted on an Olympus inverted microscope, equipped with a front-illuminated sCMOS (2560 × 2160, pixel size 6.45 μm), and an InsightSSI fluorescence illuminator, using a ×60 1.2NA water immersion objective. Filter sets were as follow: excitation 426/450 nm (cyan fluorescent protein, CFP) and 505–515 nm (YFP); emission 458/487 nm (CFP) and 520/550 nm (YFP).

#### Kymographs

Kymographs were generated using ImageJ (Multiple Kymograph plugin) averaging over a 5-pixel neighbourhood along a manual trace through the PT midline for a single or both YFP and CFP channels.

## Results and discussion

### Case study: analysis of the oscillatory behaviour of Arabidopsis pollen tubes growing *in vitro*

**Fig. 2.**
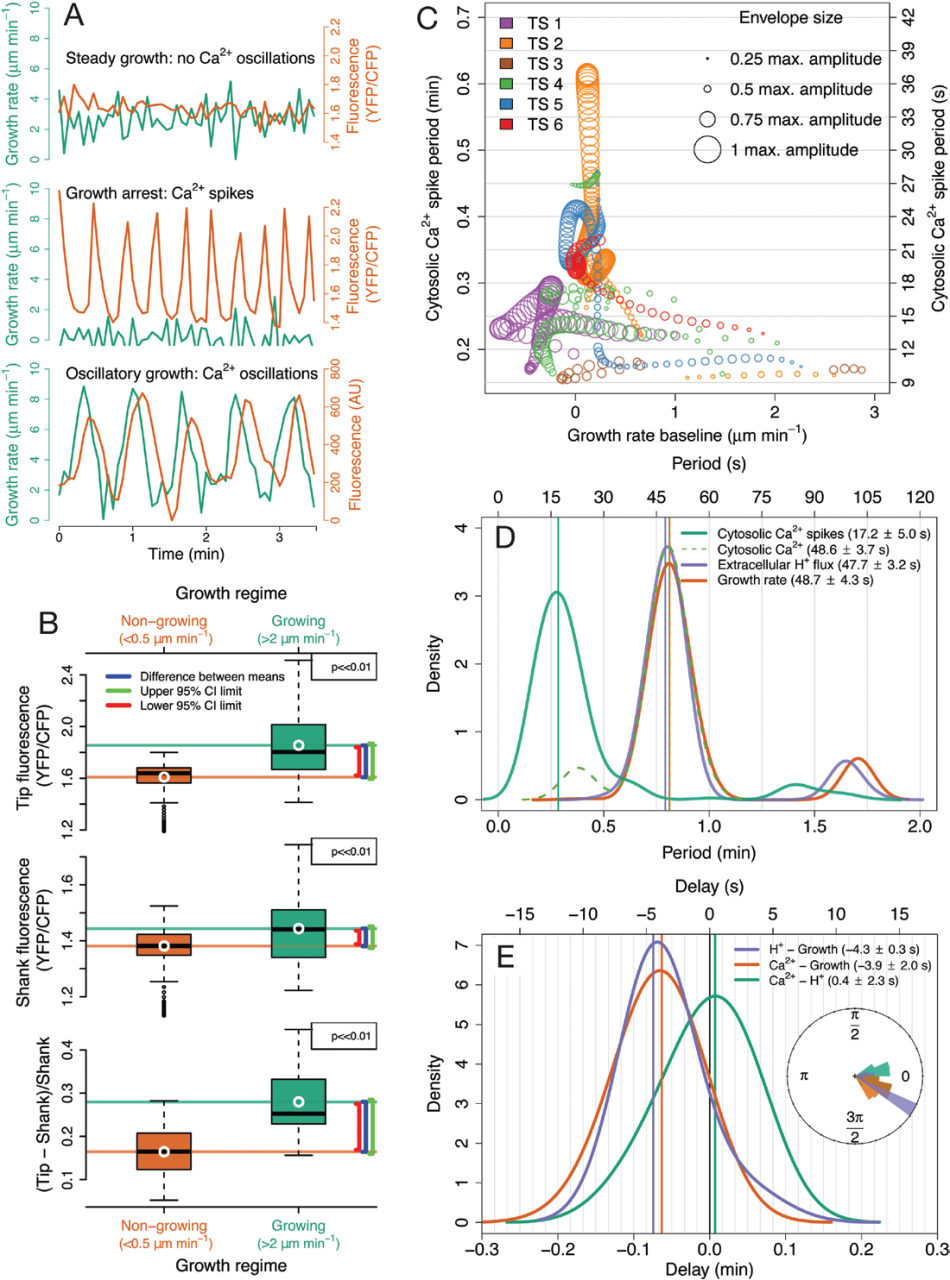
Characterization of the oscillatory features of Arabidopsis Col-0 pollen tubes growing *in vitro* by CHUKNORRIS. (**A**) Representative time series of the three growth regimes and underlying oscillatory signatures obtained from ratiometric (top and middle; from Supplementary Fig. S1-5) or single channel (bottom; from Supplementary Figs S4 and 5) kymographs. Growth rate is shown in green while fluorescence indicating Ca^2+^ concentration is shown in orange. (**B**) Differences in [Ca^2+^]_cyt_ between growing and non-growing pollen tubes measured by ratiometric fluorescence at the tip, shank, and tip/shank gradient assessed by the normalized ratio $\frac{Tip-Shank}{Shank}$ (from top to bottom) in all points of six series (Supplementary Fig. S1). White circle and solid horizontal lines show the mean, while increasing brackets represent the lower 95% confidence interval (red) of the difference between means, the measured difference (blue), and the upper 95% confidence interval (green) obtained with an unequal variances *t*-test. P-values were adjusted for multiple comparisons with Bonferroni correction. Both growth and ratiometric fluorescence are trends/baselines extracted to remove fluctuations. Growing and non-growing regimes were defined as arbitrary thresholds adequately separating the distribution of average growth (trend; Supplementary Fig. S2). (**C**) Period of Ca^2+^ spikes at the pollen tube tip as a function of average growth rate observed in six ratiometric kymographs (YFP/CFP), with the size of the circles being proportional to the maximum amplitude observed. Although pollen tubes showed growth in all time series analysed, significant periodic behaviour (extracted with a continuous wavelet transform) in the Ca^2+^ signal was mostly found in non-growing regimes. (**D**) Distributions of the period of oscillations detected in [Ca^2+^]_cyt_ spikes from ratiometric kymographs (*n* = 6), and a representative example of extracellular H^+^ flux measured together with [Ca^2+^]_cyt_ and growth rate in a single channel kymograph. Significant periods were determined with a continuous wavelet transform. Means and standard deviations were obtained from the main distribution found in a two-component Gaussian mixture model. (**E**) Distribution of delays between concomitant measurements of extracellular H^+^ influx at the tip, [Ca^2+^]_cyt_, and growth estimated by cross wavelet transform, with the inset showing a circular histogram with phase relationships obtained with the same method. Means and standard deviations were derived from the main distribution found in a two-component Gaussian mixture model.

We used CHUKNORRIS (Fig. 1) to quantify the oscillatory features of Arabidopsis PTs. So far, oscillations in growing PTs have been observed almost exclusively under *in vitro* germination conditions, described extensively in *Nicotiana*, *Petunia*, *Lilium*, and *Camellia* (Geitmann *et al.*, 1996; Geitmann and Cresti, 1998; Feijó *et al.*, 2001; Holdaway-Clarke and Hepler, 2003; Michard *et al.*, 2008). Despite some earlier data (Iwano *et al.* 2009), Arabidopsis, the best genetic system, still lacks a deep quantitative analysis of PTs oscillatory behaviour. Here, we are filling that gap by using CHUKNORRIS to characterize three distinct growth regimes in Arabidopsis Col-0 (Fig. 2). We analysed time series of growth rate, [Ca^2+^]_cyt_, and extracellular H^+^ influxes, which consistently revealed specific oscillatory signatures at the tip underlying three growth modes: (i) steady growth, (ii) growth arrest, and (iii) growth oscillations (Fig. 2A). Steady-growing PTs showed no oscillations (or undetected low amplitude oscillations) in either growth rate or [Ca^2+^]_cyt_ (Fig. 2A), with a high baseline concentration of [Ca^2+^]_cyt_ (Fig. 2B). Upon growth arrest, high amplitude oscillations in [Ca^2+^]_cyt_ occurred with high frequency (low period) and high amplitude at the PT tip (Ca^2+^ spikes; Fig. 2A, C, D), together with a decrease in the baseline cytosolic Ca^2+^ concentration (Fig. 2B). Although starting with high frequency, Ca^2+^ spikes show a pronounced drift, often reaching low frequencies (Fig. 2C). Despite the preliminary description of oscillations in Arabidopsis arrested PTs (Iwano *et al.* 2009), these were unexpected results because most *in vitro* oscillations described so far occurred exclusively in growing PTs, while all published theoretical models of PTs assume that oscillations are necessarily coupled to growth (Damineli *et al.*, 2017*b*). A third synchronous oscillatory regime also occurred, with high amplitude growth oscillations and low frequency (high period), involving oscillations with the same periodicity in [Ca^2+^]_cyt_ and extracellular H^+^ fluxes entering the tip (Fig. 2A, C). While oscillations in H^+^ fluxes and [Ca^2+^]_cyt_ were virtually simultaneous, with no delay, both appeared to lag (follow) growth oscillations by a short delay (~4 s; Fig. 2E). Furthermore, CHUKNORRIS allowed an improved characterization of the oscillations and synchronization regimes, with parameters evaluated in a time-explicit manner revealing transitions between oscillatory states.

#### Oscillation period and synchronization

Quantitative estimates of oscillation periods (Fig. 2D) computed for [Ca^2+^]_cyt_ spikes at the PT tip ($\tau_{{\left[Ca^{2+}\right]}_{cyt\;spikes}}=17.2\pm 5.0\;\text{s}$) were extracted from ratiometric kymographs (*n* = 6; individual data in Supplementary Fig. S1, available at *JXB* online). On the other hand, joint oscillations in growth rate ($\tau_{growth\;rate}=48.7\pm 4.3\;\text{s}$), extracellular H^+^ flux ($\tau_{H^{+}{}_{flux}}=47.7\pm 3.2\;\text{s}$), and [Ca^2+^]_cyt_ ($\tau_{{\left[Ca^{2+}\right]}_{cyt\;}}=48.6\pm 3.7\;\text{s}$) were derived from a single representative series, with obvious growth rate oscillations for over 20 min (Supplementary Video S1, available at *JXB* online). This sequence was used to further test the performance of the method in detecting oscillations. Periods of growth rate, H^+^ flux, and [Ca^2+^]_cyt_ oscillations reported herein were generally longer than those reported in lily (~31–42 s; Holdaway-Clarke *et al.*, 1997; Feijó, 1999; Messerli *et al.*, 1999) and shorter than those in tobacco (~78 s or ~1.3 min; Michard *et al.*, 2008). These three processes are clearly synchronized in this growth regime showing such obvious oscillations, since they showed essentially the same period and a narrow distribution of phases and delays (Fig. 2D). Growth appears to precede H^+^ flux and [Ca^2+^]*cyt* oscillations by a small delay, similar to the ~4 s reported in lily for [Ca^2+^]_cyt_ (Messerli *et al.*, 2000). In both cases, growth was determined from fluorescence images. These delays diverge significantly from the estimates obtained using growth rates determined from differential interference contrast microscopy (DIC) sequences, reported to be ~15 s for lily (Feijó, 1999; Holdaway-Clarke *et al.* 1997; Messerli *et al.*, 1999). This discrepancy highlights the importance of developing these analyses using an integrated package and fully standardized conditions for acquisition. However, a precise estimate requires greater temporal resolution, given that the time step used in measuring these variables was 4 s, not excluding the existence of even shorter delays. Even though an exhaustive characterization of these oscillations is outside of the scope of the present work, it nonetheless provides a proof of principle of the existence of such a synchronous regime and the adequacy of the methods proposed to detect them.

#### Changes in [Ca^2+^]_cyt_ and Ca^2+^ spikes

Arabidopsis PT tips show higher Ca^*2+*^ concentration than the shank, as expected by the reported tip-focused gradients (Pierson *et al.*, 1994; Holdaway-Clarke *et al.*, 1997; Michard *et al.*, 2008), with oscillations diminishing in amplitude throughout the PT (see *‘Ratiometric kymograph module’* below). Growing PTs (growth rate > 2 μm min^−1^) have significantly higher [Ca^2+^]_*cyt*_ than non-growing PTs (growth rate < 0.5 μm min^−1^) at the tip, shank, and in tip/shank gradient ($\frac{Tip-Shank}{Shank}$), regarding baseline concentrations and growth trends obtained by removing fluctuations (Fig. 2B; with tip and shank regions defined in the ‘*Ratiometric kymograph module*’ and shown in Supplementary Fig. S1). Here, we considered growth rate values that adequately separate the non-growing regime which includes very slow growth or even cytosol retracting behaviours (Supplementary Video S2, available at *JXB* online, measured as negative growth), from the growing regime, which includes steady and rapid growth (Supplementary Fig. S2, available at *JXB* online). However, the differences in [Ca^2+^]_*cyt*_ remained consistent within a wide range of thresholds used to define the growth regimes. Oscillations in tip-focused [Ca^2+^]_cyt_ associated with non-growing PTs had considerably higher frequency (shorter period) and amplitude than the oscillations that occurred simultaneous to growth oscillations (Fig. 2E). This qualitative difference warrants naming these high frequency/amplitude oscillations as Ca^2+^ spikes. This phenomenon has been reported in PTs growing closer to the embryo sac upon slowing down and arresting, having apparently compatible periods although no quantification was provided (Iwano *et al.*, 2009; Ngo *et al.*, 2014).

The relationship between Ca^2+^ oscillations and growth is central to most oscillatory models, which generally assume that they always occur together and that Ca^2+^ entry follows growth spurts (revised in Damineli *et al.*, 2017*b*). In fact, a direct correlation has been proposed between Ca^2+^ oscillations and growth (Pierson *et al.*, 1994) in all species so far described. In order to test this assumption and investigate the relationship between Ca^2+^ oscillation frequency and growth rate, the period and amplitude of Ca^2+^ spikes detected were shown as a function of average growth rate (Fig. 2C). Interestingly, high amplitude spiking (shown by larger circles) did not rely on high growth rates, with a longer period being associated with slower growth. The relationship is not linear and reveals a pronounced frequency drift, with single bouts of spikes (contiguous circles) changing periods over 2-fold. The scarcity of detectable oscillations during growth is contrasted with their profusion in slow-growing or growth-arrested PTs, shedding considerable doubt on the necessity of growth spurts to elicit Ca^2+^ oscillations (Supplementary Fig. S1). The quantification of [Ca^2+^]_cyt_ spikes frequency in Arabidopsis (Col-0) is novel and not immediately comparable to estimates of regular [Ca^2+^]_cyt_ oscillations during growth, as the former show a pronounced change in frequency through time and the latter is absent in the data set of ratiometric kymographs we have processed so far (*n* > 20).

CHUKNORRIS revealed a much more complex oscillatory pattern than predicted by any of the models previously described (Damineli *et al.*, 2017*b*). If all PTs have a conserved mechanism of growth related to Ca^2+^, as the structural evidence seems to favour, than none of the published models capture the nature of the relationship between growth and Ca^2+^. These results also challenge the assumptions that Ca^2+^ entry primarily occurs through stretch activated channels opened due to membrane tension during growth, as well as the notion that oscillations in growth and [Ca^2+^]_cyt_ are necessarily coupled (Damineli *et al.*, 2017*b*). That is not to say that Ca^2+^ and growth are unrelated, especially during high amplitude growth oscillations, but it is as treacherous to derive causality from temporality as to invoke causation from correlation (Damineli *et al.*, 2017*b*). Instead, there appears to be specific frequency signatures underlying distinct growth regimes, with fast spiking during slow/growth-arrested PTs and longer periods in oscillatory-growing PTs (Fig. 2A–E). However, the synchronization properties of different cellular processes within PTs are unlikely to be simple and their understanding requires adequate quantitative approaches, as presented herein.

### Features of the CHUKNORRIS analysis pipeline

While the previous section focused on the practical application of our method to Arabidopsis PTs, we now detail the different modules, test their robustness, and propose possible applications. CHUKNORRIS was tailored to tackle what we considered the two main constraints to the quantitative analysis of oscillations in PTs and other moving cells: (i) resolution of growth time series, and (ii) methods to assess the time-varying frequency/amplitude of oscillations, as well as their phase relationships/delays.

The general workflow of CHUKNORRIS is described in Fig. 1, which depicts each of the five analysis modules presented and their integration (corresponding R scripts can be found in Supplementary Data S1).

#### Tip detection module: subpixel growth rate and oscillations

**Fig. 3.**
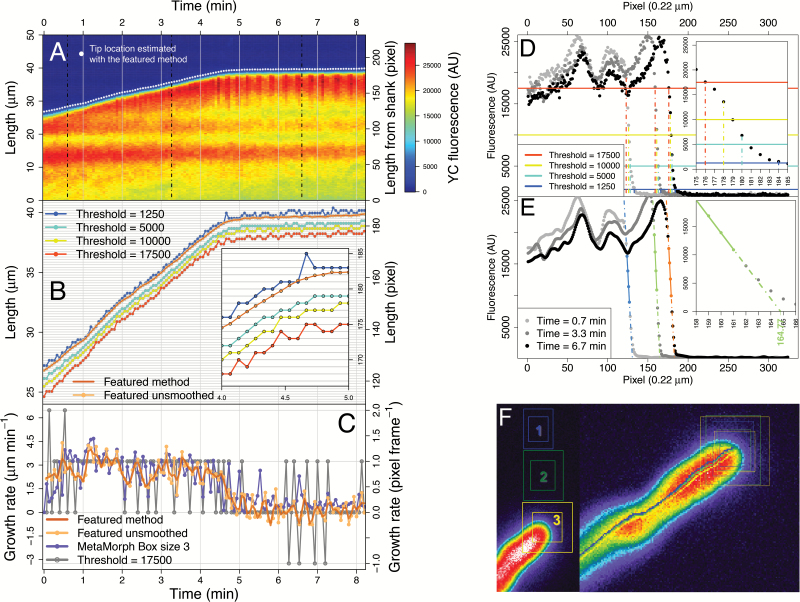
Tip detection: comparison between methods in an illustrative kymograph. (**A**) Kymograph of the YFP channel showing the tip location detected by CHUKNORRIS (featured method). Dotted lines are the time points used in D and E. (**B**) Tip location estimates compared between CHUKNORRIS and a simple threshold method. Inset shows a ‘zoom in’ evidencing subpixel resolution, as the detection of CHUKNORRIS is not limited to whole pixels. (**C**) Growth rate time series obtained from the threshold, MetaMorph, or CHUKNORRIS (featured method). (**D**) Example of tip detection by the threshold method with three time points indicated in A. (**E**) Example of tip detection by CHUKNORRIS with the same three time points indicated in A but with a smoothed fluorescence series. (**F**) MetaMorph region of interest of three different sizes (left) used for simultaneous tracking (right).

A major limitation in achieving a quantitative understanding of oscillations in PTs is the precision in which growth rate is measured. Despite many sophisticated methods for tip finding, currently there is a shortage of straightforward approaches to generate growth rate time series with subpixel resolution without relying on commercial software or intricate model-fitting procedures, which can still perform insufficiently for this purpose. Here, a novel method based on linear regression is compared to other standard practice, that is, using a threshold in fluorescence to detect cell boundaries (in this case the PT tip), both of which are applied to kymographs. These methods were then compared to the industry reference package, MetaMorph, which features a semi-automatic template-matching algorithm in the ‘Track Object’ function (Fig. 3), a principle employed in diverse tracking software less often used in PTs (Masuzzo *et al.*, 2016), motivating the choice of MetaMorph for comparison.

A kymograph generated in a standard image analysis software (e.g. ImageJ) features in each line the fluorescence profile of the object of interest (PT tip in this case) and the background, corresponding to a time point, with growth being visualized through time with the stack of lines (Fig. 3A; lines across space are vertical while fluorescence across time corresponds to horizontal lines). The simplest approach to detect the tip, or any other cell boundary, would be to consider a threshold in fluorescence above which the object is distinguished from the background. The pixel in which this threshold is exceeded corresponds to the estimate of the tip location, per time point (Fig. 3D). Obtaining this estimate for all times yields a time series of pixels in which the tip is supposed to be located according to the threshold chosen (Fig. 3B). Thus, the resolution of a detection method solely based on a fluorescence threshold is limited to whole pixels and shows discrete values, most often implying disjoined fluctuations in the corresponding growth rate series (Fig. 3C).

In contrast, CHUKNORRIS employs a subpixel tip detection method using linear regression. When observing the fluorescence profile at each time point of a kymograph, a sharp increase is evident at the tip when looking from background to the object (Fig. 3E). This increase has a near-linear part spanning several pixels that can be used to extract information and predict the tip location, without being limited to discrete pixels. Our approach is to fit a linear model to the sharpest slope and use the estimated intercept of the pixel grid as a proxy for tip location (Fig. 3E). The fit is performed in a lightly smoothed version of the fluorescence profile, for each time point, to avoid noise in the automatic choice of the sharpest slope. This algorithm generates a series of tip location estimates that is not constrained to discrete pixels (Fig. 3B). Consequently, the velocity series obtained has subpixel resolution and is a smoother function as compared to the threshold method (Fig. 3C). The estimates of tip location can be smoothed with a local polynomial fit assuming that there are no ‘sudden jumps’ of the tip location. While smoothing reduces noise, it also decreases the amplitude of high frequency oscillations or even abolishes them. Thus, ideally only small portions of the time series (span) should be used in the local polynomial fit.

**Fig. 4.**
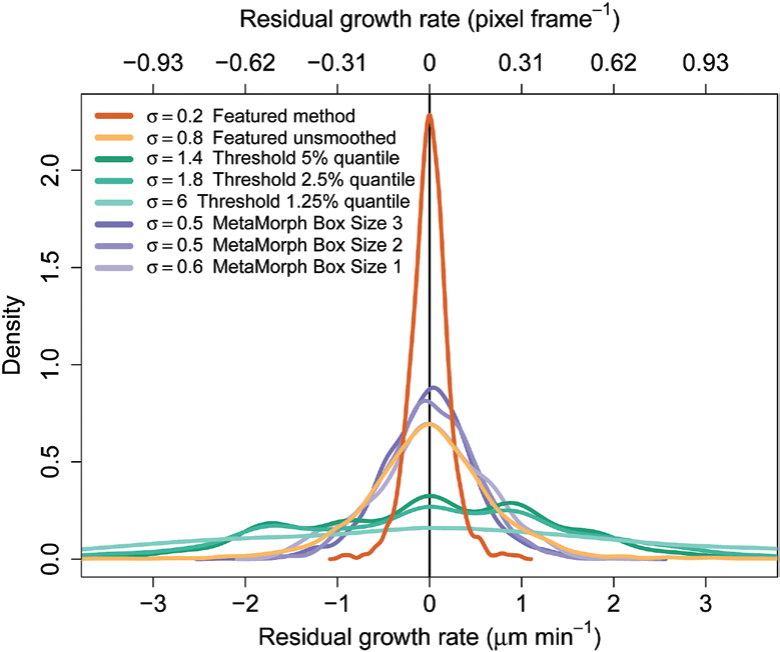
Noise estimates of different tip detection methods for a standard data set. Density of high frequency components (the noise band) was extracted from growth rate series estimated in a standard data set of kymographs (*n* = 6) with threshold, MetaMorph, or CHUKNORRIS (featured method).

**Fig. 5.**
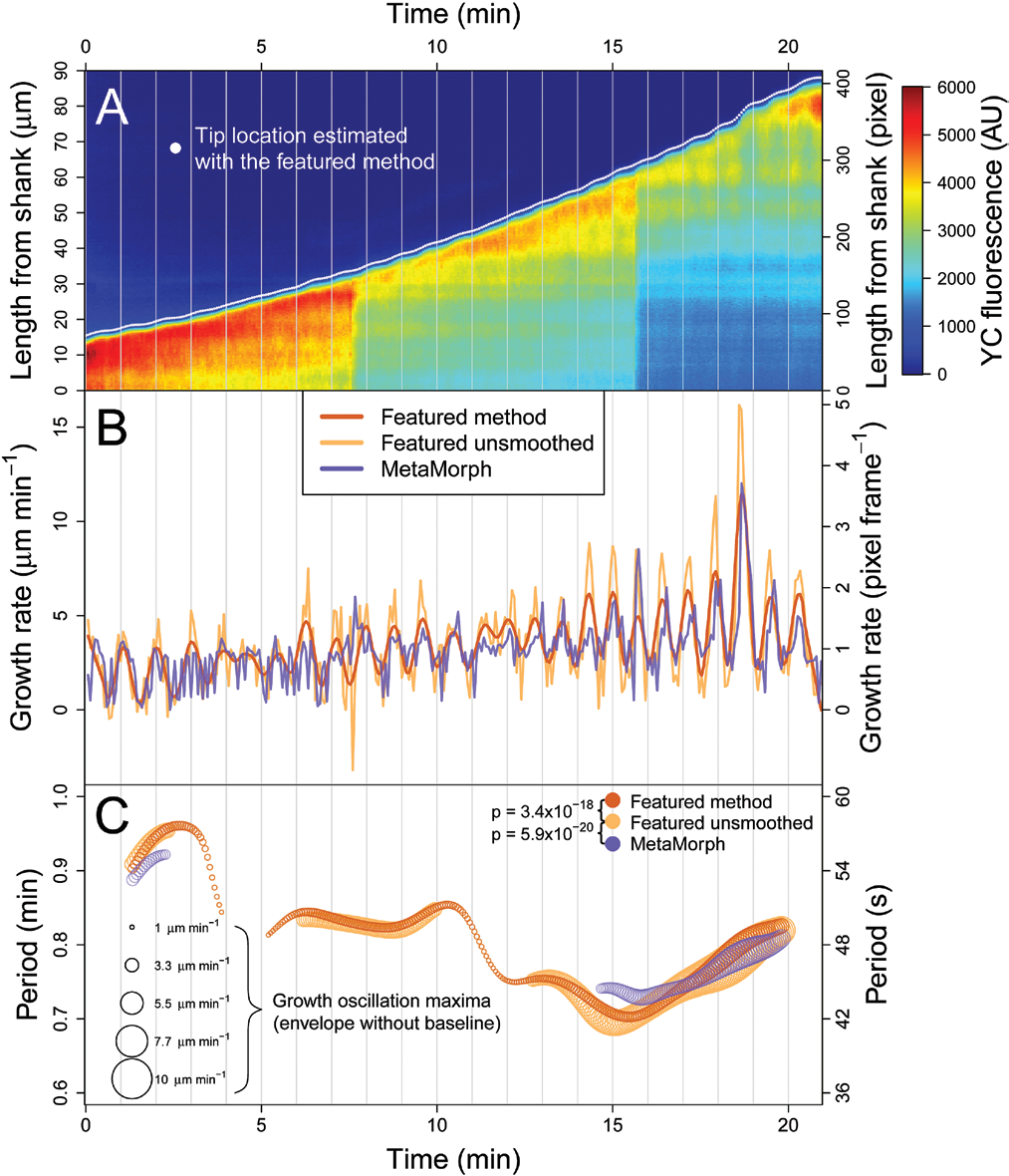
Comparing the detection of periodic components. (**A**) Oscillatory single channel kymograph with tip location detected by CHUKNORRIS (featured method). (**B**) Growth rate series estimated by MetaMorph or CHUKNORRIS (featured method) smoothed or not. (**C**) Period of oscillations detected in the series above by the different methods, with the diameter of the circles being proportional to the amplitude of the growth oscillation detected (envelope without the baseline/trend). Periodic components of the detrended time series in C obtained with a continuous wavelet transform. The smooth version of CHUKNORRIS clearly outperforms the other alternatives. Significant differences in detecting oscillations were accessed with a paired proportion test (McNemar’s) on 2 × 2 contingency tables.

CHUKNORRIS was then compared with MetaMorph, whose template-matching algorithm also yields velocity series with subpixel resolution (Fig. 3C). The analysis is made upon selection of a rectangular region of interest, which is searched in the specified vicinity in the next frame, finding the location that most closely matches the chosen template (Fig. 3F)—a similar approach to many popular algorithms (Messerli *et al.*, 1999; Masuzzo *et al.*, 2016). Importantly, the MetaMorph algorithm processes considerably more information than CHUKNORRIS, because it extracts the position from a 2D image instead of 1D rows of the kymograph. Although the *a priori* assumption would be a significant loss precision from using only 1D, CHUKNORRIS surprisingly not only produced a comparable amount of noise (Fig. 4), but also showed a greater ability to detect oscillations under the circumstances tested (Fig. 5). Additionally CHUKNORRIS is fast, it is independent of user intervention, which avoids the introduction of artefacts in the tracking process, and it provides arbitrary smoothness. The comparison was extended to three MetaMorph box sizes, chosen to have object regions of 500 (25 × 20; 23.33 μm^2^), 750 (30 × 25; 34.99 μm^2^), and 900 (30 × 30; 41.99 μm^2^) pixels, with search regions of 1200 (40 × 30; 55.99 μm^2^), 2000 (50 × 40; 93.31 μm^2^), and 2500 (50 × 50; 116.64 μm^2^) pixels, respectively. Tracking with all methods was performed in all samples of the data set presented here (*n* = 6), with their resulting noise shown in Fig. 4 and individual series in Supplementary Fig. S1 panels D_i_–F_i_. Noise was evaluated by isolating the fast frequencies in each series using the multiresolution analysis introduced in the “Time-series analysis module” section below. The tracking noise appears to decrease with increasing box size for MetaMorph, being lower although still comparable with the unsmoothed version of CHUKNORRIS (Fig. 4). All subpixel methods are more precise and less biased than threshold methods, with the smoothed version of CHUKNORRIS having the lowest noise (Fig. 4).

The ability of each method to detect oscillations was then investigated using a long and highly oscillatory time series (Fig. 5A, Supplementary Video S1). In order to guarantee an impartial comparison, the kymograph used in the analysis was extracted from a modified version of the tracking trace generated on MetaMorph. The series of tip location estimates generated by the ‘Track Object’ routine (line in Supplementary Video S1) was extended to create the line used to extract pixel intensities at each time point, yielding the kymograph. This procedure controls for noise and biases caused by manually drawing paths along the PT midline, which is the traditional way to generate kymographs. Visually it is clear that the unsmoothed method of CHUKNORRIS captures oscillations more consistently than MetaMorph, with the smoothed version improving the detection even further (Fig. 5B). The detection of oscillations *per se* was evaluated as the proportion of time a significant periodic component was present in the continuous wavelet spectrum (introduced in the “Time-series analysis module” section below), which provides a time-explicit estimate of period and amplitude (Fig. 5C). The figure shows the period of oscillations detected for each time point, with circle size being proportional to the amplitude (more precisely, the envelope of the oscillations). The smoothed version of CHUKNORRIS captures significantly more oscillations than the unsmoothed version, despite the lower amplitude, with both versions detecting significantly more than MetaMorph. Significance was determined by McNemar’s proportion test for paired samples applied to a 2 × 2 contingency table (Supplementary Table S1, available at *JXB* online).

#### Ratiometric kymograph module: fluorescence time series along the pollen tube

**Fig. 6.**
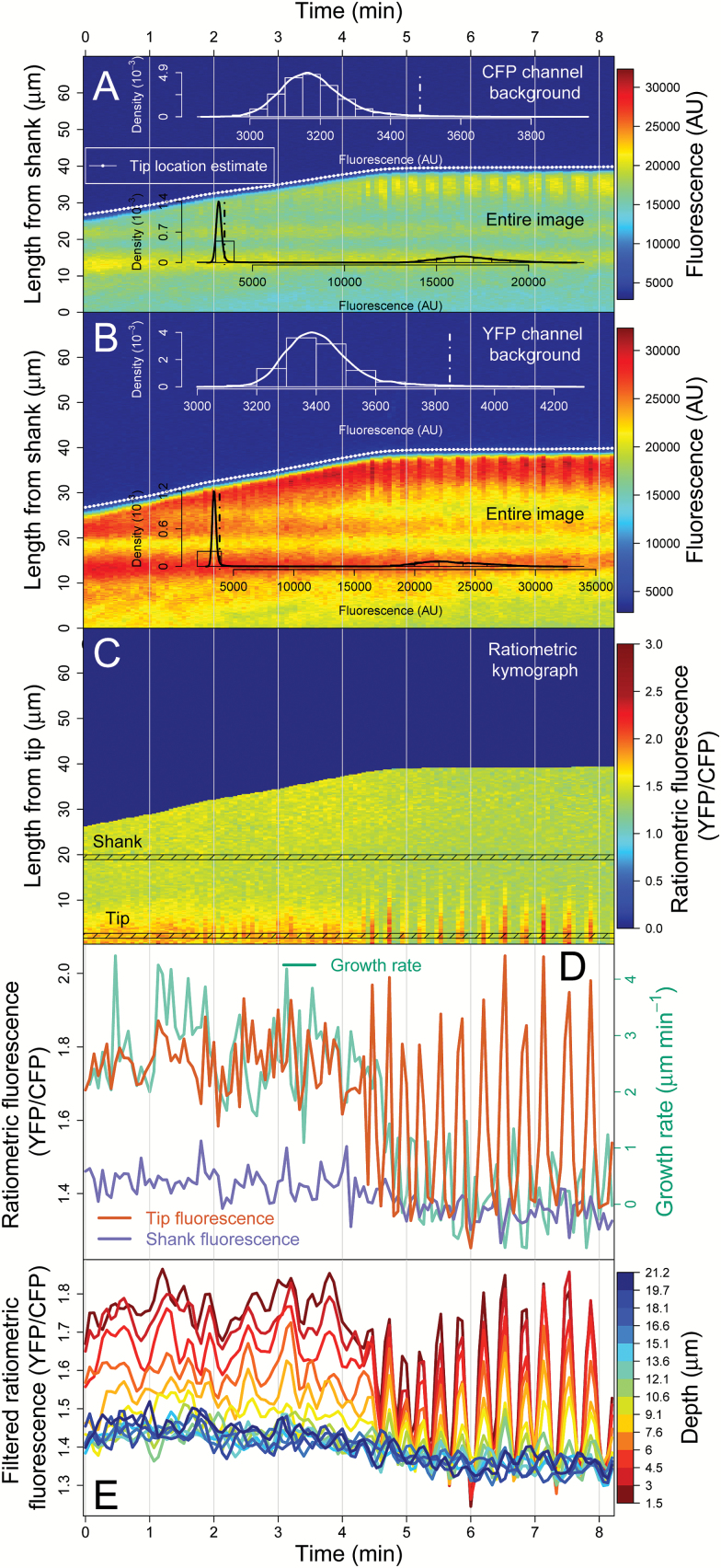
Ratiometric kymograph module. (**A**) Kymograph of the CFP channel showing the distribution of background fluorescence in the white inset, determined from the tip location estimated by CHUKNORRIS. The cut-off at the 99% quantile is shown in the dotted line in the fluorescence background and entire image (black inset). (**B**) Kymograph of the YFP channel showing the same information as in A but with a clearly different background level. (**C**) Ratiometric kymograph obtained after subtracting the background of each channel $\frac{YFP-YFP_{Background}}{\text{CFP}-{\text{CFP}}_{Background}}$ and aligning kymographs by the tip location estimate, which allows the extraction of fluorescence time series throughout the pollen tube. Tip and shank series were averaged in the corresponding highlighted regions. (**D**) Tip and shank ratiometric fluorescence series (indicating [Ca^2+^]_cyt_) obtained from the tip aligned kymograph together with growth rate. (**E**) Ratiometric fluorescence time series throughout the pollen tube averaged in ~1.1 µm regions at ~1.5 µm intervals, from tip to shank with noise filtered for clarity.

CHUKNORRIS can also calculate the distribution of background fluorescence (noise) and selectively subtract it from each channel based on the estimates of tip location acquired on the previous module. While in Fig. 3 the analysis was restricted to a single channel, two kymographs can be generated from ratiometric probes, here CFP and YFP (Fig. 6A, B). The estimates of tip location performed in the strongest channel (in this case, YFP in Fig. 6B) were used in both channels to distinguish the region containing the fluorescence signal from the background fluorescence. Considering an arbitrary quantile in the tail of the distribution as a cut-off (here, 99%), CHUKNORRIS achieved adequate separation between the distributions of background and signal, allowing the subtraction of a specific value for each channel (dotted lines in the inset histograms Fig. 6A, B) and then calculation of the ratio $\frac{\;Channel_{YFP}-Background_{YFP}}{Channel_{CFP}-Background_{CFP}}$. To facilitate the analysis of fluorescence time series, each kymograph row was flipped at the estimate of tip location aligning the tip on the same side of the matrix (Fig. 6C). The resulting kymograph has all time points at the same relative distance from the estimated tip location. This procedure enables the selection of an arbitrary distance from the tip and extraction of a fluorescence time series, in this case ratiometric, considering a given width for averaging. Here, we compare the fluorescence series at the tip versus the shank, defined as the median of a region ~1.1 μm wide at ~2.2 μm (tip) and ~19.4 μm (shank) from the tip estimate, together with growth rate (Fig. 6D; definition used in Supplementary Fig. S1). Then, oscillations throughout the PT were analysed from ~1.5 μm to ~21.5 μm from the tip, in regions ~1.1 μm wide and ~1.5 μm apart, evidencing a decrease in amplitude and basal levels of fluorescence from tip to shank (Fig. 6E; series filtered as detailed in the “Time-series analysis module” section below).

The Kymograph module analyses a single channel kymograph in a similar manner. However, while ratiometric kymographs tend to correct acquisition artefacts, these are often difficult to remove in only one channel and can compromise the reliability of the fluorescence time series obtained. For example, while growth oscillations are evident in Fig. 5A, an oscillatory fluorescence signal is barely visible because the kymograph is contaminated by artefacts such as refocusing events and photobleaching. The single channel kymograph module is illustrated in Supplementary Fig. S3 (available at *JXB* online) featuring a 2D version of a discrete wavelet transform, the same type of filtering used for time series, which will be described in the next section. It is able to remove trends and kinks in the image to an extent that enables the recovery of the oscillatory fluorescent signal at the tip, which is only partially visible by naked eye in the movie (Supplementary Video S1). Oscillations in Ca^2+^ extracted from this single channel kymograph are then analysed with other concomitant variables (Supplementary Fig. S4, available at *JXB* online), as discussed in the “Synchronization module” section.

#### Time-series analysis module: extracting time-resolved periodic components

A generic time series may have a heterogeneous sampling rate, be contaminated with outliers, have a variable baseline and heterogeneous frequency/amplitude of oscillations. To address these issues, the time-series analysis module of CHUKNORRIS features three parts: (i) a pre-filtering module, (ii) the main frequency-specific filter, and (iii) a time-resolved analysis of the frequency/amplitude of the oscillations. The application of this module is illustrated for a time series of electrophysiology data (extracellular H^+^ flux measurements at the PT tip), but could be used in any other time series, including imaging (e.g. the fluorescence series from the kymograph modules).

The pre-filtering module is used to ensure homogeneity in sampling rate and to remove outliers (Supplementary Fig. S5, available at *JXB* online). Most methods of time-series analysis rely on a homogeneous sampling frequency, that is, all time steps must be the same. Yet many experimental methods have significant variation in the interval of time between samples. In this algorithm, the time interval between measurements is calculated and a new time grid with regular intervals is created. The time series is then interpolated between the original time points sampled by *loess*, a local polynomial regression fitting (Supplementary Fig. S5A) (Cleveland and Devlin, 1988). This method allows time-series values to be estimated for the new regular time grid (Supplementary Fig. S5B), even in the presence of missing values. In addition, outliers of different types are expected to contaminate most time series (Chen and Liu, 1993). Although gross outliers can easily be detected with a simple criterion, as the points deviating from a given amount of the median absolute deviation (Iglewicz and Hoaglin 1993), in live-cell time series, outliers are usually much harder to correct, making the use of specific packages crucial (López-de-Lacalle, 2016). The performance of this procedure was illustrated by inserting four artificial outliers in a series of extracellular H^+^ flux measurements, with two adding and two subtracting 10 pmol cm^−2^ s^−1^ to the original values; these were successfully detected and restored to values lying close to the original data (Supplementary Fig. S5C).

**Fig. 7.**
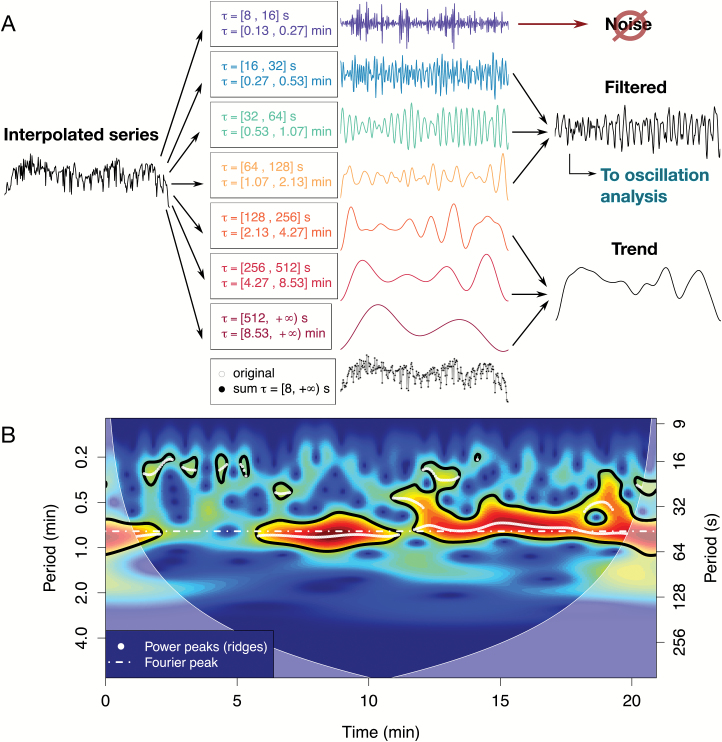
Time-series analysis module. (**A**) Filter using a discrete wavelet transform. After interpolation and outlier removal, the time series is divided into different levels of details corresponding to different frequency bands (with periods indicated with τ). The highest frequency band contains noise and can often be discarded to provide smoothing. The low frequency band corresponds to trends and baseline changes, which have to be subtracted for further analysis. The middle bands, where the signal of interest usually lies, are combined, yielding a filtered series where oscillations are analyzed. (**B**) Periodicity analysis with a continuous wavelet transform. Colours correspond to the power of specific components through time (time-frequency space) with significant (*P* < 0.05) periods circled in black. White dots indicate the peaks of the power spectrum detected (wavelet ridges), while the white dotted line shows the frequency peak of the Fourier spectrum for comparison. The shaded regions correspond to the ‘cone of influence’, a region in which analysis is not reliable.

The main filtering step is performed by decomposing a time series in different levels of detail, conferring frequency-specificity to the output. Specific behaviour falls in defined frequency bands that when summed together yield the original signal (Fig. 7A). While high frequency bands contain high details, corresponding to fast fluctuations and often noise, low frequency bands contain coarser details, including changes in baseline (trend), which must be removed to correctly analyse the period/amplitude of the oscillations. Usually, the signal of interest lies in the middle frequency bands, which would contain the periodic components of biological significance; however, including the noise frequency band can be relevant to analyse fast spiking behaviour. This is performed with a discrete wavelet transform (Fig. 7A; R package ‘wavelets’; Aldrich, 2013). It essentially acts as a band pass filter in which discrete frequency bands are selected or discarded, providing smoothing when discarding high frequency and de-trending when eliminating low frequency. Importantly, it allows the composition of a resulting filtered series containing only the frequencies of interest for further analysis. For example, the high frequency bands are removed in series in which they do not contain information (e.g. the analysis of the series in Fig. 5B comprised only periods from 16 to 128 s), while they are included in the analysis of high frequency spikes (e.g. the series used in Fig. 2C included shorter periods, spanning from 8 to 128 s).

Finally, after ensuring homogeneous sampling rate and filtering the desired periodic components, frequency and amplitude can be estimated. Given that the frequency and amplitude of oscillations of cellular processes can vary in time (non-stationary signal), a continuous wavelet transform (package ‘biwavelet’ by Gouhier *et al.*, 2016) was used instead of Fourier or auto-correlation analysis, given that the last two assume that period and frequency are constant over time. In rough terms, wavelet analysis decomposes a time series into the time-frequency space by measuring its correlation with a single wave of a specific form for all time points and for different sizes of this wavelet, which correspond to different frequencies and amplitudes (Torrence and Compo, 1998). The wavelet (here of Morlet type) is shifted across time by changing a location parameter while its frequency and amplitude are changed with a scale parameter, which dilates or compresses it. A measure of the correlation for each location and scale, the wavelet coefficient, is then used to produce the wavelet spectrum depicting the power of different regions of the time-frequency space (Fig. 7B). Significance, shown in Fig. 7B with regions surrounded by black lines, is tested using the null model that the spectrum of a stochastic process only depends on its previous state, like Brownian motion (Grinsted *et al.*, 2004). Peaks in the statistically significant parts of the power spectrum, the so-called wavelet ridges (shown in Fig. 7B with white dots), are then extracted with a custom peak-finding algorithm, which allows further analysis of the dominant frequencies and amplitudes. The distribution of periods in the wavelet ridges detected in the data set presented are summarized in Fig. 2D.

#### Synchronization module: joint periodic components, phase relationships, and delays

**Fig. 8.**
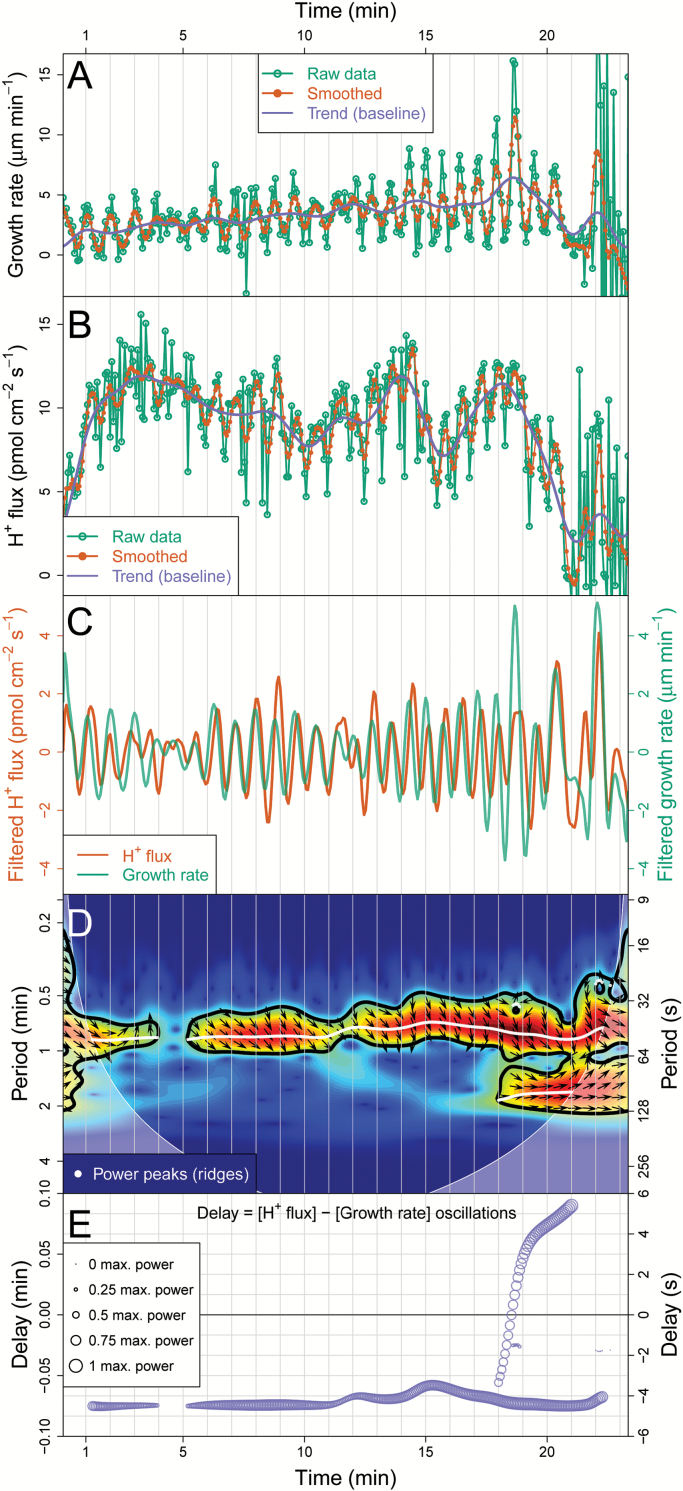
Synchronization analysis module. (**A**) Growth rate series extracted from a single channel kymograph with the corresponding smoothing and extracted trend. (**B**) Extracellular H^+^ fluxes at the tip (influx) measured concomitantly with the series in A, with its corresponding smoothing and extracted trend. (**C**) Matched and filtered series (using a discrete wavelet transform). (**D**) Cross wavelet transform showing regions of significant joint periodicity with arrows indicating the phase relationship. The arrows point to the direction of the trigonometric circle corresponding to the local phase angle between two series (e.g. *x* and *y*), both being in phase if arrows point to the right, in anti-phase if pointing to the left, *x* precedes *y* if pointing up, while *y* precedes *x* if pointing down (further information in the main text under “Synchronization module”). Colours correspond to the power of specific components through time (time-frequency space) with significant (*P* < 0.05) periods circled in black. White dots indicate the peaks of the power spectrum detected (wavelet ridges), while the shaded regions correspond to the ‘cone of influence’, a region in which analysis is not reliable. (**E**) Time delay estimated with the cross wavelet analysis between extracellular H^+^ flux and growth rate oscillations, showing that growth leads (occur before) by a small delay for most of the series, until a long-period component appears (seen in D) introducing another delay. The size of the circles shows the relative oscillation amplitude (normalized by maximum power).

Synchronization is a fundamental process in biology, although not always trivial to detect (Glass, 2001). CHUKNORRIS was developed to assess the synchronization between two time series recorded simultaneously, even if their relationship changes in time. In order to illustrate a time-explicit evaluation of synchrony, this module was applied to the growth rate series extracted from imaging (kymograph Fig. 5A) with the corresponding series of extracellular H^+^ flux measurements at the tip of the same cell (Fig. 8). Both time series were measured simultaneously, but they stem from independent experimental methods. CHUKNORRIS matches the raw data of each series to a common time frame, performing interpolation as needed, removing trend and noise (Fig. 8A, B). The resulting series pair clearly displays joint behaviour (Fig. 8C), even towards the end of this series where one would normally discard the data because the PT completely ceases growth, showing the often-reported ‘balloon’ shape (Supplementary Video S1).

Joint periodicity, phase relationship, and delay between two time series were then analysed, using a method similar to the continuous wavelet analysis. The simplest approach is to perform a wavelet analysis on each series and compare their significant periods, amplitudes, and phases. This is basically the procedure of a cross wavelet analysis, revealing areas in the time-frequency spectrum with high common power (Grinsted *et al.*, 2004; statistically more robust alternatives can also be used, such as wavelet coherence). The cross wavelet spectrum for extracellular H^+^ flux and growth rate is shown in Fig. 8D, with arrows depicting their phase relationship. Phase relationships are especially relevant to establish whether the time series are synchronized, which occurs when there is a constant phase difference through time. The arrows indicate the local phase angle between two series, pointing to the corresponding direction of the trigonometric circle (e.g. *x* and *y* with phase difference $\Delta \varphi =\varphi_{x}-\varphi_{y}$). Two series are in phase if arrows point to the right (Δ*φ* = 0), in anti-phase if pointing to the left (Δ*φ* = π), with *x* coming before *y* if pointing up (phase leading by $\Delta \varphi =\frac{\pi }{2}$) and *x* coming after *y* if pointing down (phase lagging by $\frac{\pi }{2}$, that is $\Delta \varphi =-\frac{\pi }{2}\;OR\;\frac{3\pi }{2}$) (Fig. 8D). Furthermore, the phase relationships estimated at specific frequencies allow the delay between oscillations at that frequency to be calculated, providing an estimate of time-varying delays. In Fig. 8E the delay is approximately maintained until the PT starts to acquire the balloon shape, when suddenly another strong long-period component appears (Fig. 8D). This change drives the delay from the negative to the positive region (Fig. 8E). Even if the oscillations continue to be roughly in phase because the delays are small, this example reveals the potential of the method. In essence, CHUKNORRIS unveils an apparent change in the temporal order of the two measured variables (growth and H^+^ influx) concomitant with a morphogenetic change leading to growth arrest, which has never been detected using other methods. Given all the literature in a field that elaborates on causality based on temporal sequence, this example alone reveals the importance of high-precision quantitative methods of analysis.

#### Synchronization in multiple cellular processes

Finally, synchronization can be assessed in all three variables acquired concomitantly, that is, extracellular H^+^ flux, growth, and fluorescence, by using the kymograph module and filtering presented in Supplementary Fig. S3. The filtered fluorescence signal of probe YC3.6 shows a clear oscillatory signal at the tip (Supplementary Video S1 and Fig. S4A), corresponding to [Ca^2+^]_cyt_ oscillations. After removing the trend (Fig. 8A, B and Supplementary Fig. S4A), all three variables can be seen to oscillate with remarkably similar periods (Supplementary Fig. S4B), confirmed by the cross wavelet analysis between [Ca^2+^]_cyt_ and extracellular H^+^ flux (Supplementary Fig. S4C) and [Ca^2+^]_cyt_ and growth rate (Supplementary Fig. S4D). The delays between all variables are quantified through time (Supplementary Fig. S4E), showing narrow distributions summarized in Fig. 2D, E together with the phase relations, supporting that in this regime all variables are synchronized.

### Limitations of CHUKNORRIS in analysing oscillations in single cells

The use of high fluorescence thresholds to detect the tip in kymographs evidences an issue with inferring growth from fluorescence signals alone (Fig. 3B). The nature of fluorescence and growth becomes ambiguous since higher fluorescence usually implies apparent growth. Both CHUKNORRIS and MetaMorph detect growth oscillations in seemingly growth-arrested PTs, requiring further studies to disentangle the effect of high amplitude Ca^2+^ spikes and low amplitude growth rate oscillations.

Single channel kymographs (Fig. 5A) can show a series of artefacts that are normally corrected in the ratiometric case (Fig. 6A–C). This includes differences in focus that can occur when PTs change planes, potentially leading to fluorescence blocks as in Fig. 5A. In addition, ‘kinks’ and ‘bulges’ may form naturally in PTs, leading to a local fluorescence increase, potentially seen as horizontal stripes in the shank regions as in Fig. 3A. These large blocks, stripes, and kinks can be removed or ameliorated with filtering (Supplementary Fig. S3) but at a risk of affecting the accuracy of the final parameters derived.

In addition, detecting statistically significant low amplitude oscillations can be challenging in the presence of high peaks, as they bias the power of the wavelet spectrum and hence the significance test. Reconstructing a time series from significant periods detected, subtracting the reconstructed signal from the original series, and re-analysing iteratively may circumvent this limitation.

## Conclusion

This work quantitatively characterizes three distinct oscillatory regimes in Arabidopsis PTs, including steady growth with no detectable oscillations, growth arrest with high frequency Ca^2+^ spikes, and oscillatory growth with synchronized oscillations of cytosolic Ca^2+^ and extracellular H^+^ flux. Besides being suggestive of new hypotheses on growth mechanisms, these findings challenge the generalized view that Ca^2+^ primarily enters the PT tip as a response to over-growth, pointing to a more complex regulatory system involving ion dynamics.

Achieving these results was possible with CHUKNORRIS, an analysis pipeline that in our appreciation constitutes an important step towards a more complete qualitative and quantitative understanding of the oscillatory phenomena in growing or moving cells. Although developed to investigate oscillations in PTs and their relationship with growth, CHUKNORRIS is immediately extensible for other cell types showing tip growth or cellular movement in general. Proper characterization of biological oscillations demands time-explicit methods such as the ones employed here, as these oscillations are complex and often show varying frequency and amplitude, and can even transition between synchronization regimes. Furthermore, the diversity of data types and analysis methods hinders comparison between different works, which adds to the difficulty of attaining estimates to adequately test specific hypotheses. Consequently, mechanistic studies and accurate phenotyping capable of detecting subtle effects of mutations, as in ion channels and transporters, on the oscillatory behaviour of single cells demand tools such as CHUKNORRIS.

## Supplementary data

Supplementary data are available at *JXB* online.

Data S1: Code, data, and supporting material including a tutorial and examples.

Table S1: Contingency tables comparing tip detection methods.

Video S1: Oscillatory growth with tip location trace.

Video S2: Pollen tube showing negative growth and oscillations.

Figure S1: Individual ratiometric kymographs used in this work.

Figure S2: Distribution of average growth rates.

Figure S3: Filtering of a single channel kymograph.

Figure S4: Synchronization between [Ca^2+^]_cyt_, H^+^ flux, and growth rate oscillations.

Figure S5: Pre-filtering module showing interpolation and outlier removal.

## Data deposition

Data from all six ratiometric kymographs with corresponding MetaMorph tracking, as well as ion flux, tracking, and kymograph data of the highly oscillatory growth series are available at Dryad Digital Repository: http://dx.doi.org/10.5061/dryad.6806c. Code in the statistical programming language R, together with examples of its usage are available in the online repository GitHub: https://github.com/damineli/CHUKNORRIS, last accessed 15 February 2017.

## Supplementary Material

supplementary_dataset_S1

supplementary_video_S1

supplementary_video_S2

supplementary_Table_S1_Figures_S1_S5

## Abbreviations:

[Ca2+]_*cyt*_: cytosolic calcium concentration
CYP: cyan fluorescent protein
PT: pollen tube
YFP: yellow fluorescent protein.
