# Supplementary material for: Oscillatory signatures underlie growth regimes in Arabidopsis pollen tubes: computational methods to estimate tip location, periodicity, and synchronization in growing cells
Source: J Exp Bot. 2017 Mar 28;68(12):3267–81. doi: 10.1093/jxb/erx032 (PMC5853864; doi:10.1093/jxb/erx032)
Supplement: supplementary_dataset_S1 [file erx032_suppl_supplementary_dataset_s1.zip › CHUKNORRIS-master/README.html]

# CHUKNORRIS

Computational Heuristics for Understanding Kymographs and aNalysis of Oscillations Relying on Regression and Improved Statistics

The code and tutorial are still on alpha version 0.1, so try to tame CHUKNORRIS with `Tutorial.Rmd` / `Tutorial.pdf` or contact the author at damineli@umd.edu

## Disclaimer

The code is provided “as is” and will unceremoniously crash or provide spurious results if used improperly.
