# Supplementary material for: Oscillatory signatures underlie growth regimes in Arabidopsis pollen tubes: computational methods to estimate tip location, periodicity, and synchronization in growing cells
Source: J Exp Bot. 2017 Mar 28;68(12):3267–81. doi: 10.1093/jxb/erx032 (PMC5853864; doi:10.1093/jxb/erx032)
Supplement: supplementary_dataset_S1 [file erx032_suppl_supplementary_dataset_s1.zip › CHUKNORRIS-master/Tutorial.html]

# Taming `CHUKNORRIS`

## A tutorial on Computational Heuristics for Understanding Kymographs and aNalysis of Oscillations Relying on Regression and Improved Statistics

Author: Daniel S.C. Damineli

Contact e-mail: damineli@umd.edu

Date: February 15, 2017

Version: 0.1.0

## Summary

This is the first version of a tutorial attempting to tame `CHUKNORRIS`, a collection of R functions designed to estimate tip location, periodicity, and synchronization in growing cells. There are 4 main modules that serve as entry points to the analyses with corresponding exemplified R scripts that can be modified to suit different scenarios. In summary, a generic time series can be analyzed with the `TimeSeriesAnalysis` yielding a time-explicit estimate of oscillatory characteristics as period, phase and amplitude. The synchronization of a pair of time series can be assessed with the `SynchronizationAnalysis` module that provides estimates of significant commom periodic components, phase relationships and delays. Single kymographs are analyzed with the `KymographAnalysis` module that provides estimates of apical growth with tip location, growth and fluorescence series. Finally, quantitative analysis in ratiometric kymographs with two distinct fluorescence channels can be perfomed with the `RatiometricKymographAnalysis` module, which enables background subtraction of each specific channel achieving a more robust indicator of fluorescent reporters. It also makes use of other modules and outputs an array of useful time series and analyses. The example codes serve as a hands-on tutorial while this document is simply an inital guide. Please visit the GitHub site for updates: https://github.com/damineli/CHUKNORRIS

## Requirements

1. Install R (developed under version 3.3.2): https://cran.r-project.org/
2. Install RStudio (developed under version 1.0.44): https://www.rstudio.com/
3. Learn the basics of R language if you are not familiar, you will thank me! (further down the road)
   - For an easy, fun bur inflexible introduction tryR: http://tryr.codeschool.com/
   - For a great tool swi`r`l: http://swirlstats.com/
4. Download GitHub CHUKNORRIS clone (if you have’t already): https://github.com/damineli/CHUKNORRIS

## Getting started

1. Placing the `CHUKNORRIS` folder in your desired path
2. Open RStudio and choose the analysis modules that suits your needs
   - One of 4 in `./CHUKNORRIS/R/`
   - Feel free to explore any other functions within the subfolders of `./CHUKNORRIS/R/src/` if you know what to do with them
3. Within the analysis module be sure to specify all itens that says `# *** MUST SPECIFY! ***` by its side
4. Make sure you follow the file format requirements
5. Run the entire script when finished e.g. `Code > Run Region > Run All`
   - Make sure you check for shortcuts to run it e.g. `cmd + A` followed by `cmd + ENTER` on MacOSX
6. Check ouput folders and run again and to find appropriate values for key parameters

## Analyzing oscillations with `RunTimeSeriesAnalysis.R`

1. Script located at `./CHUKNORRIS/R/`
2. Input: a generic time series in `.csv` format
   - example input from `./CHUKNORRIS/R/data/vp/`
3. Main parameters: explained in the file
4. Output: 2 `.csv` tables and 1 multi-page `pdf`
   - example output in `./CHUKNORRIS/R/out_exs/time_series/`
   - oscillatory characteristics in `OscillationAnalysisTbl` and `OscillationAnalysis` (figures)
   - summary statistics in `SummaryStats`

## Analyzing synchronization with `RunSynchronizationAnalysis.R`

1. Script located at `./CHUKNORRIS/R/`
2. Input: two time series in `.csv` format
   - example input from `./CHUKNORRIS/R/data/vp/` and `./CHUKNORRIS/R/data/tack/only_ts/`
3. Main parameters: explained in the file
4. Output: 3 types of `.csv` tables and 2 types of multi-page `pdf`
   - example output in `./CHUKNORRIS/R/out_exs/sync/`
   - oscillatory characteristics of both series in `OscillationAnalysisTbl` and `OscillationAnalysis` (figures)
   - summary statistics of both series in `SummaryStats`
   - synchronization estimates as joint periodicity, phase relationship and delays in `SyncTbl` and `Sync` (figures)

## Analyzing kymographs with `RunKymographAnalysis.R`

1. Script located at `./CHUKNORRIS/R/`
2. Input: a single matrix of pixel intensities in `.txt` format
   - check file for more requirements of the input format
   - example input from `./CHUKNORRIS/R/data/kymo/`
3. Main parameters: explained in the file
4. Output: 4 types of `.csv` and 2 types of multi-page `pdf`
   - example output in `./CHUKNORRIS/R/out_exs/kymo/`
   - original kymograph with estimates of tip location in `Kymo` (figure)
   - tip-aligned kymograph in the `.csv` table `TipAlignedKymo`
   - extracts time series of growth rate and fluorescence along the tube `KymoTimeSeries`
   - oscillatory characteristics of all series in `OscillationAnalysisTbl` and `OscillationAnalysis` (figures)
   - summary statistics for all series in `SummaryStats`

## Analyzing ratiometric kymographs with `RunRatiometricKymographAnalysis.R`

1. Script located at `./CHUKNORRIS/R/`
2. Input: Two matrices of pixel intensities from different fluorescence channels in `.txt` format
   - check file for more requirements of the input format
   - example input from `./CHUKNORRIS/R/data/ratio_kymo/`
3. Main parameters: explained in the file
4. Output: 4 types of `.csv` and 2 types of multi-page `pdf`
   - example output in `./CHUKNORRIS/R/out_exs/ratio_kymo/`
   - original kymograph with estimates of tip location and tip aligned ratiometric kymograph in `RatioKymo` (figure)
   - background subtracted and tip-aligned ratiometric kymograph in the `.csv` table `TipAlignedKymo`
   - extracts time series of growth rate and fluorescence along the tube `RatioKymoTimeSeries`
   - oscillatory characteristics of all series in `OscillationAnalysisTbl` and `OscillationAnalysis` (figures)
   - summary statistics for all series in `SummaryStats`
