# Supplementary material for: Oscillatory signatures underlie growth regimes in Arabidopsis pollen tubes: computational methods to estimate tip location, periodicity, and synchronization in growing cells
Source: J Exp Bot. 2017 Mar 28;68(12):3267–81. doi: 10.1093/jxb/erx032 (PMC5853864; doi:10.1093/jxb/erx032)
Supplement: supplementary_dataset_S1 [file erx032_suppl_supplementary_dataset_s1.zip › CHUKNORRIS-master/out_exs/kymo/OscillationAnalysis_TIP.FLUO_Arab_Col-0_Kymo_190815-A.txt.pdf]

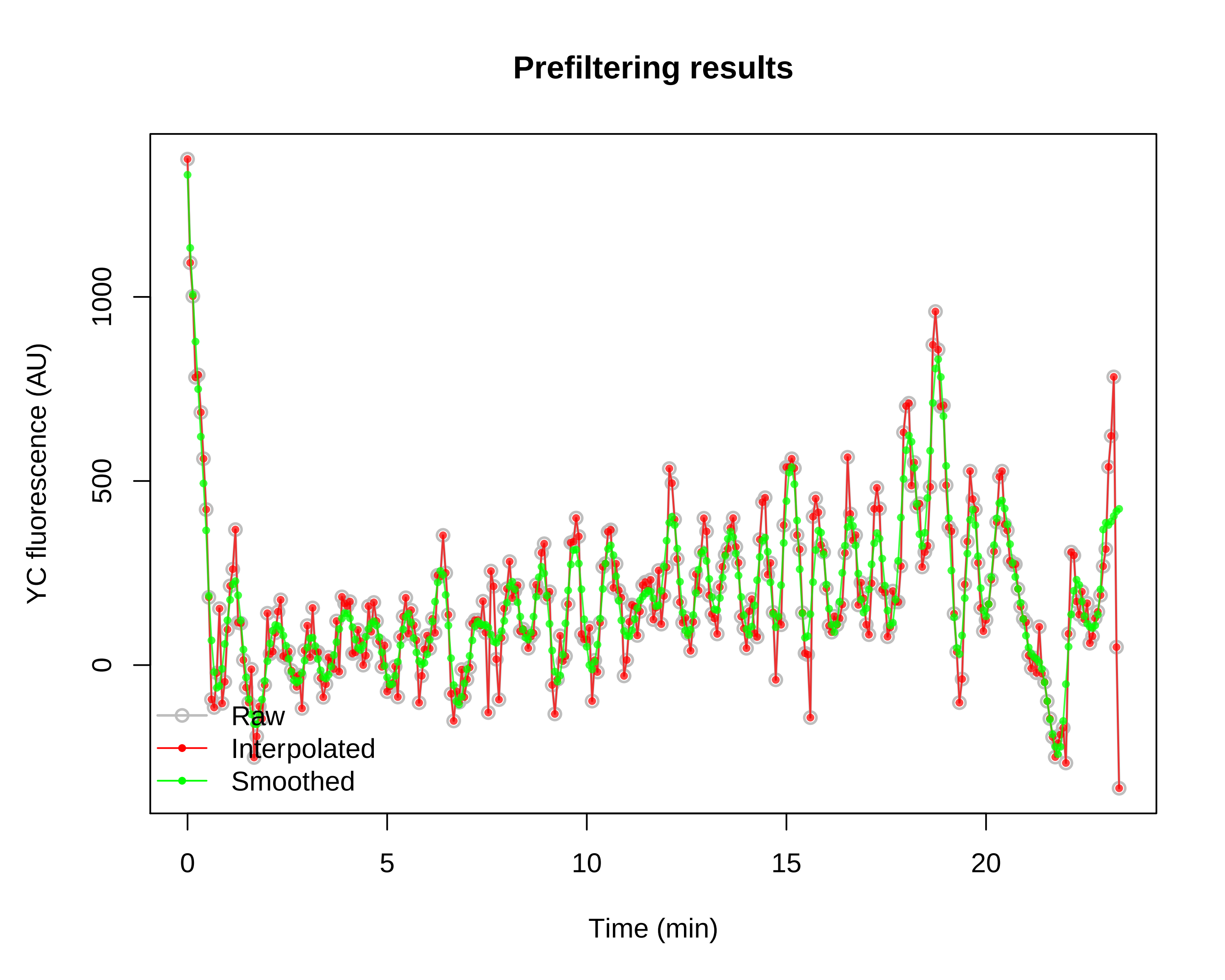
Prefiltering results
YC fluorescence (AU)
Time (min)
0
500
1000
0
5
10
15
20
Raw
Interpolated
Smoothed

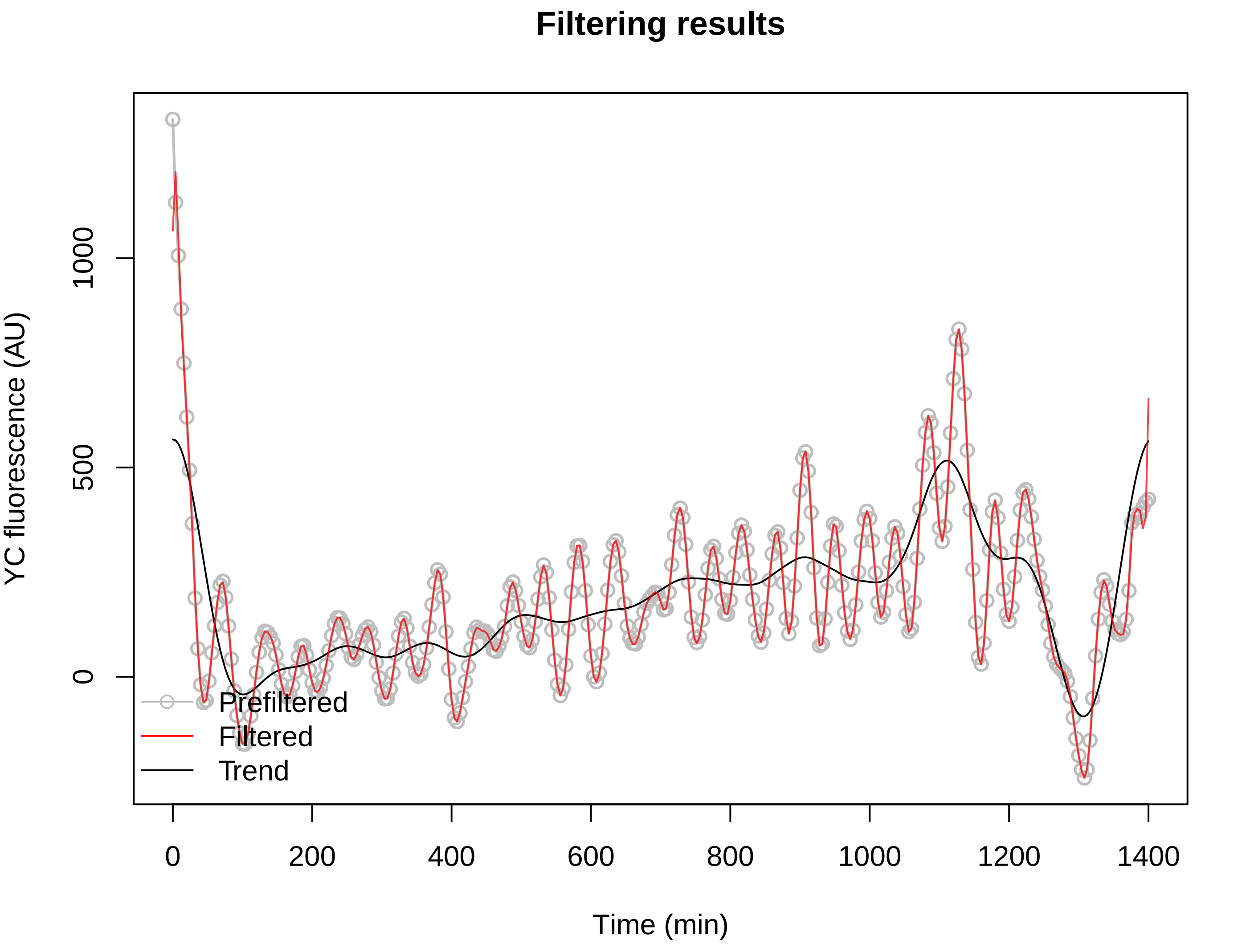

Filtering results
YC fluorescence (AU)
Time (min)
0
500
1000
0
200
400
600
800
1000
1200
1400
Prefiltered
Filtered
Trend

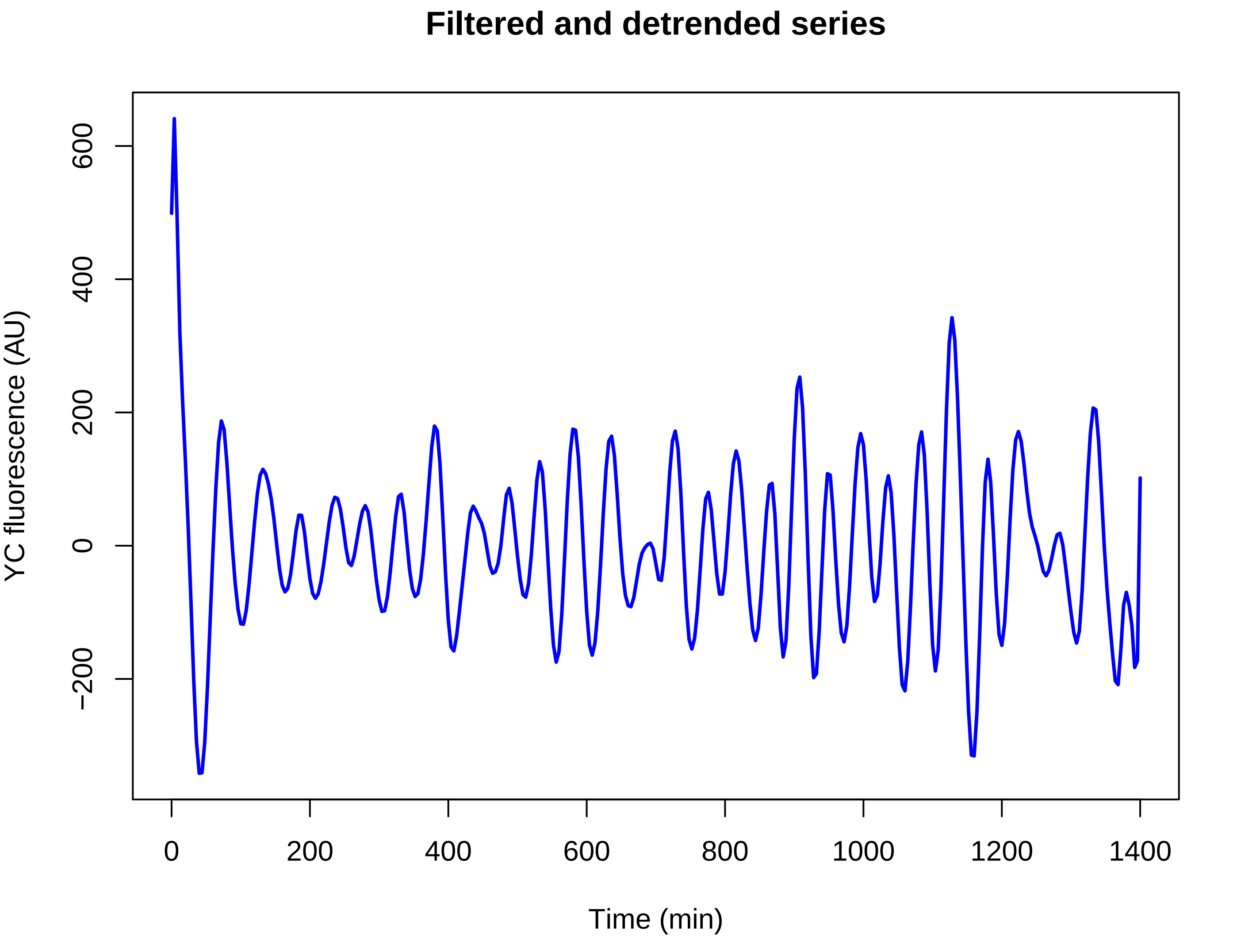
Filtered and detrended series
YC fluorescence (AU)
600
400
200
0
−200
0
200
400
600
800
1000
1200
1400
Time (min)

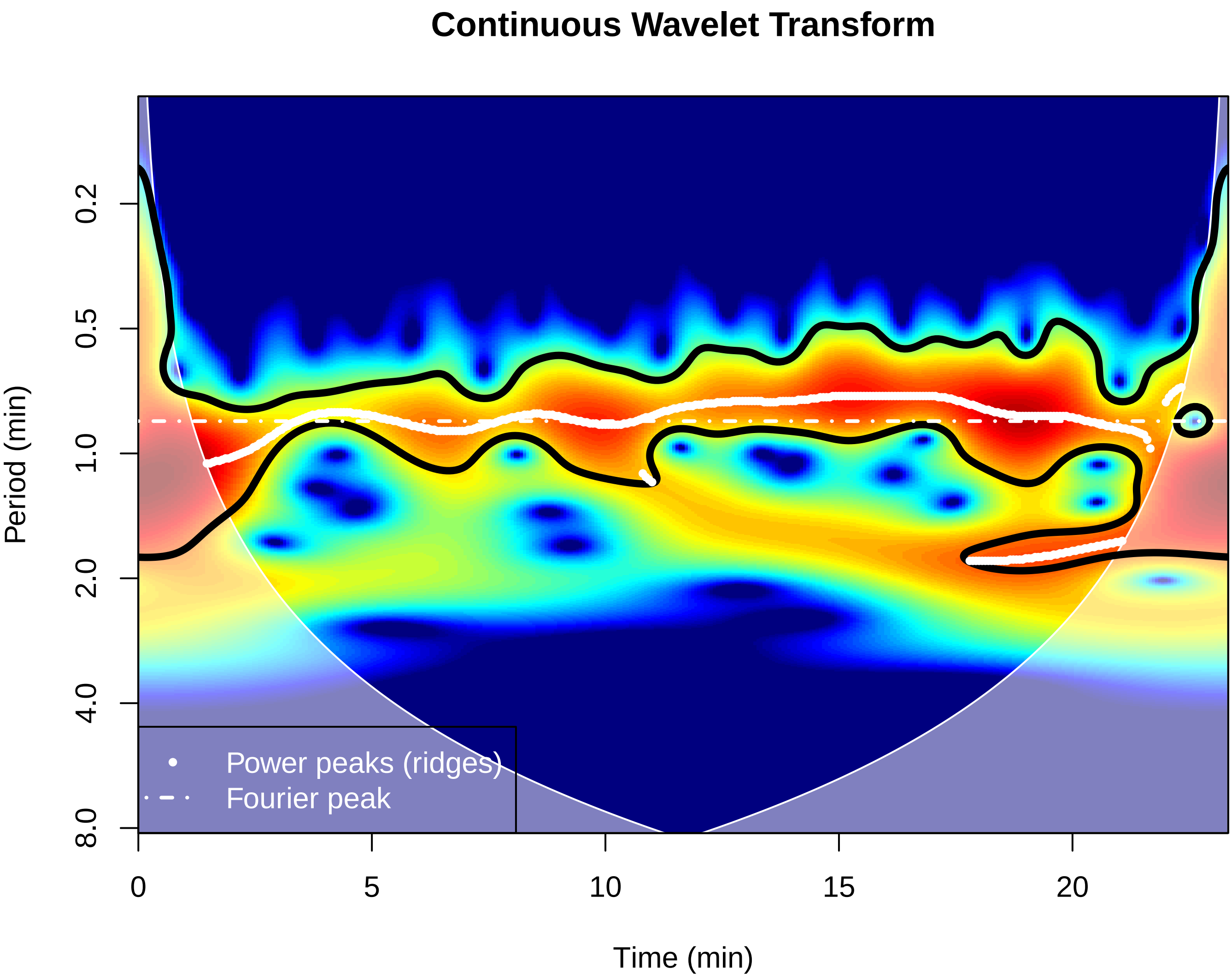

Continuous Wavelet Transform
Period (min)
0.2
0.5
1.0
2.0
4.0
8.0
Time (min)
0
5
10
15
20
Power peaks (ridges)
Fourier peak
