# Supplementary material for: Oscillatory signatures underlie growth regimes in Arabidopsis pollen tubes: computational methods to estimate tip location, periodicity, and synchronization in growing cells
Source: J Exp Bot. 2017 Mar 28;68(12):3267–81. doi: 10.1093/jxb/erx032 (PMC5853864; doi:10.1093/jxb/erx032)
Supplement: supplementary_dataset_S1 [file erx032_suppl_supplementary_dataset_s1.zip › CHUKNORRIS-master/out_exs/ratio_kymo/OscillationAnalysis_GROWTH.RAW_Arab_Col-0_Kymo_020813-A_CFP.txt.pdf]

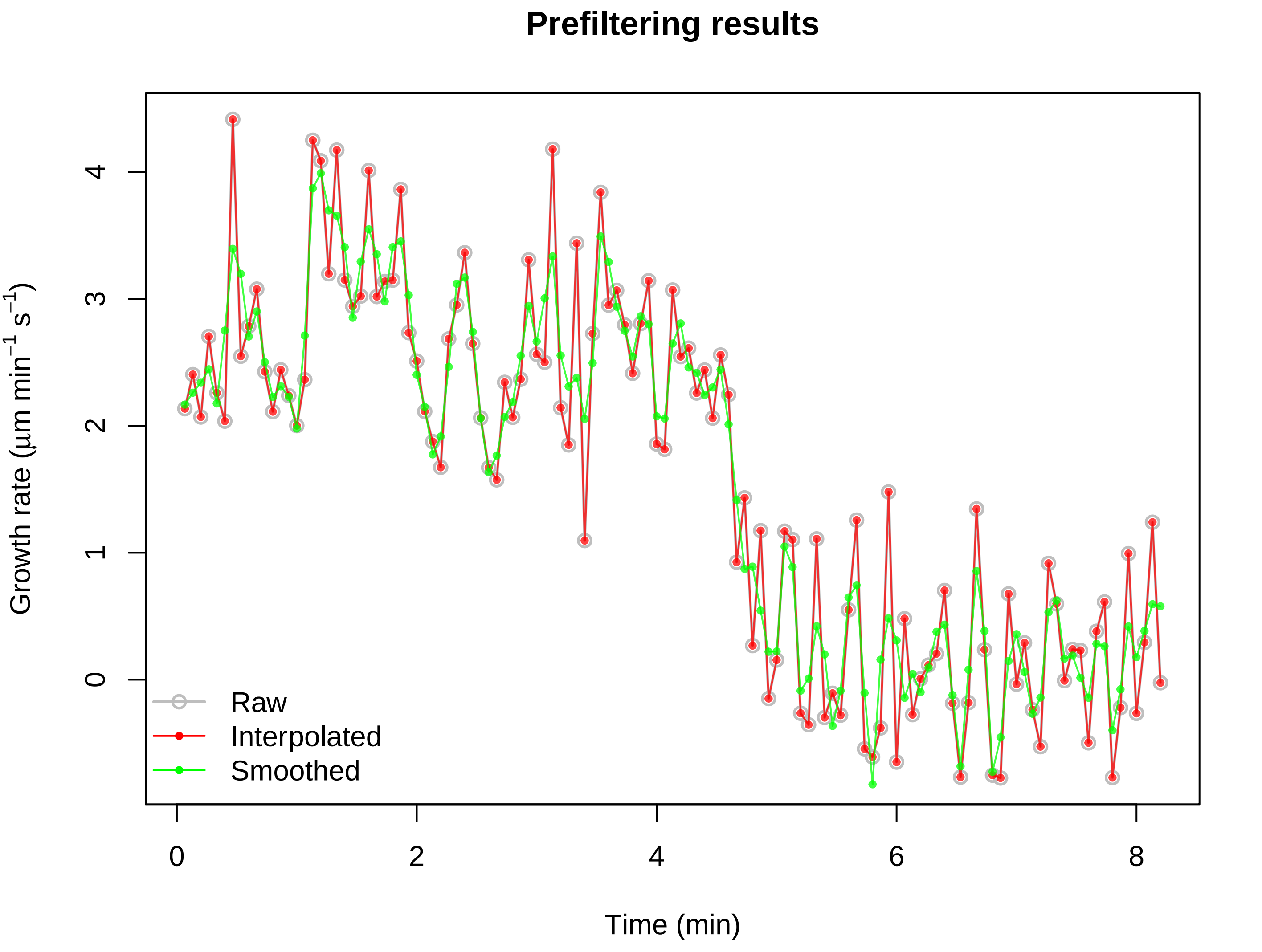

Prefiltering results
Growth rate (µm min^{-1} s^{-1})
Time (min)
Raw
Interpolated
Smoothed
0
1
2
3
4
0
2
4
6
8

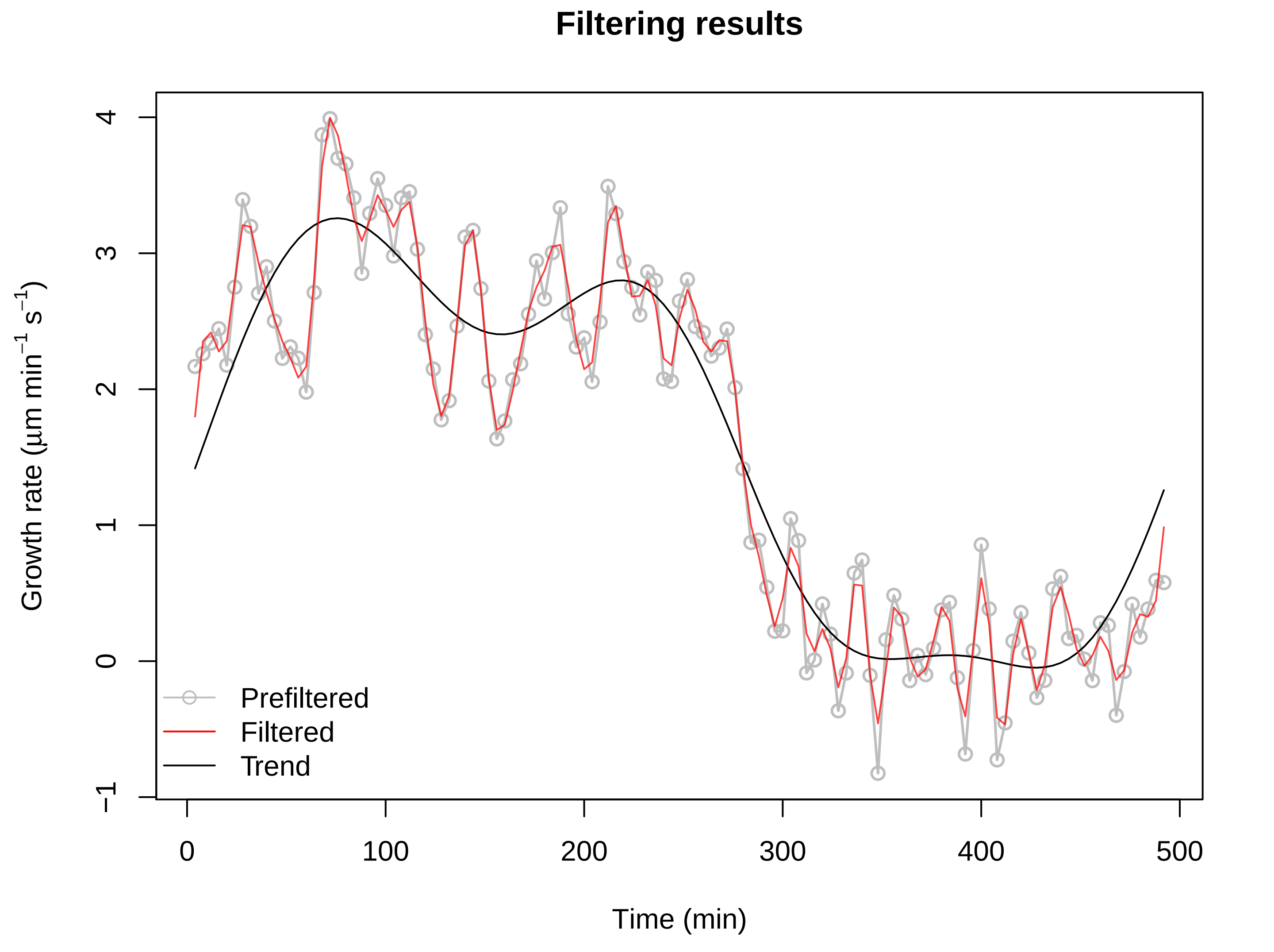
Filtering results
Prefiltered
Filtered
Trend
Growth rate (µm min^{-1} s^{-1})
Time (min)
−1
0
1
2
3
4
0
100
200
300
400
500

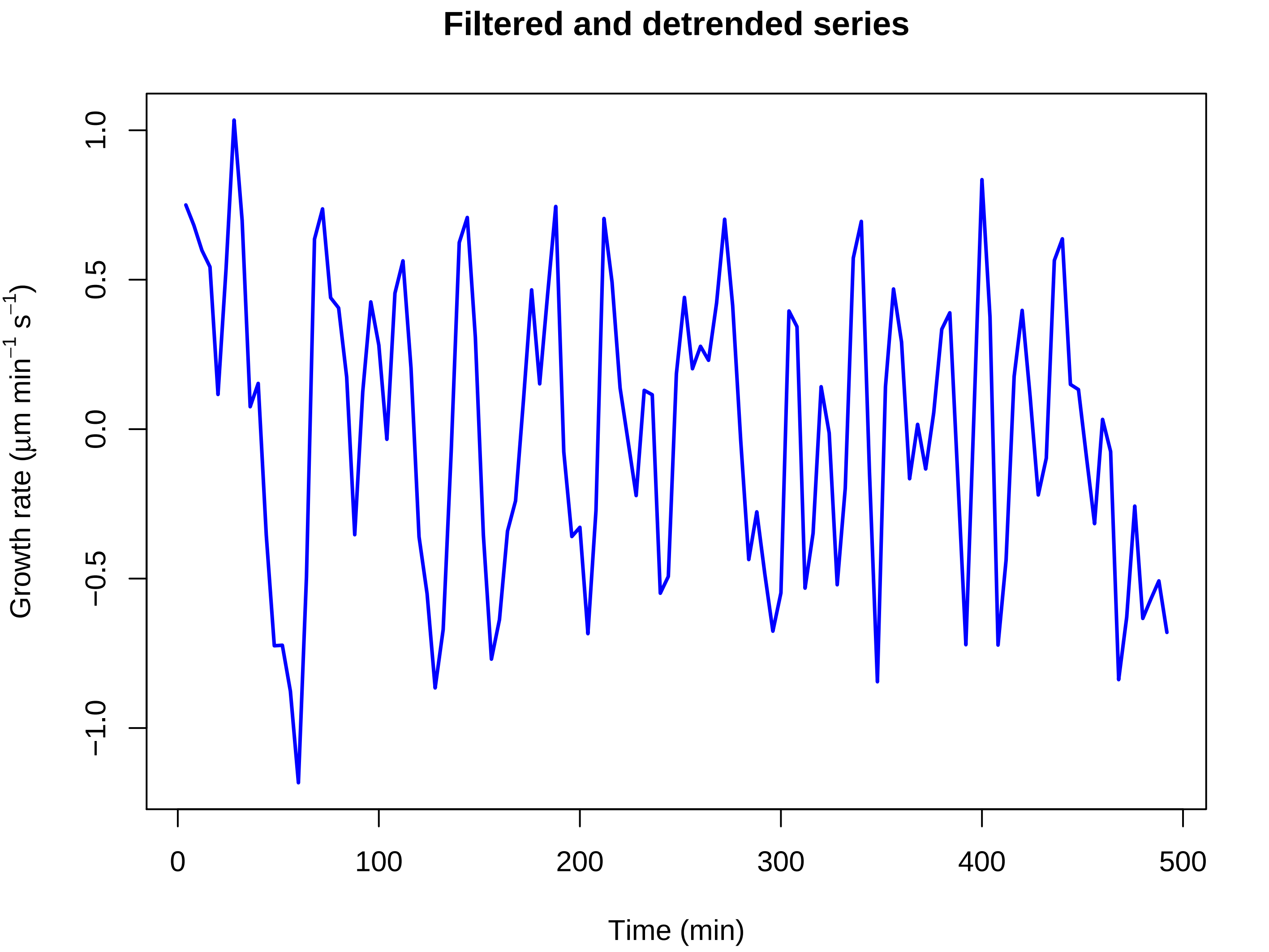
Filtered and detrended series
Growth rate (μm min−1 s−1)
1.0
0.5
0.0
−0.5
−1.0
0
100
200
300
400
500
Time (min)

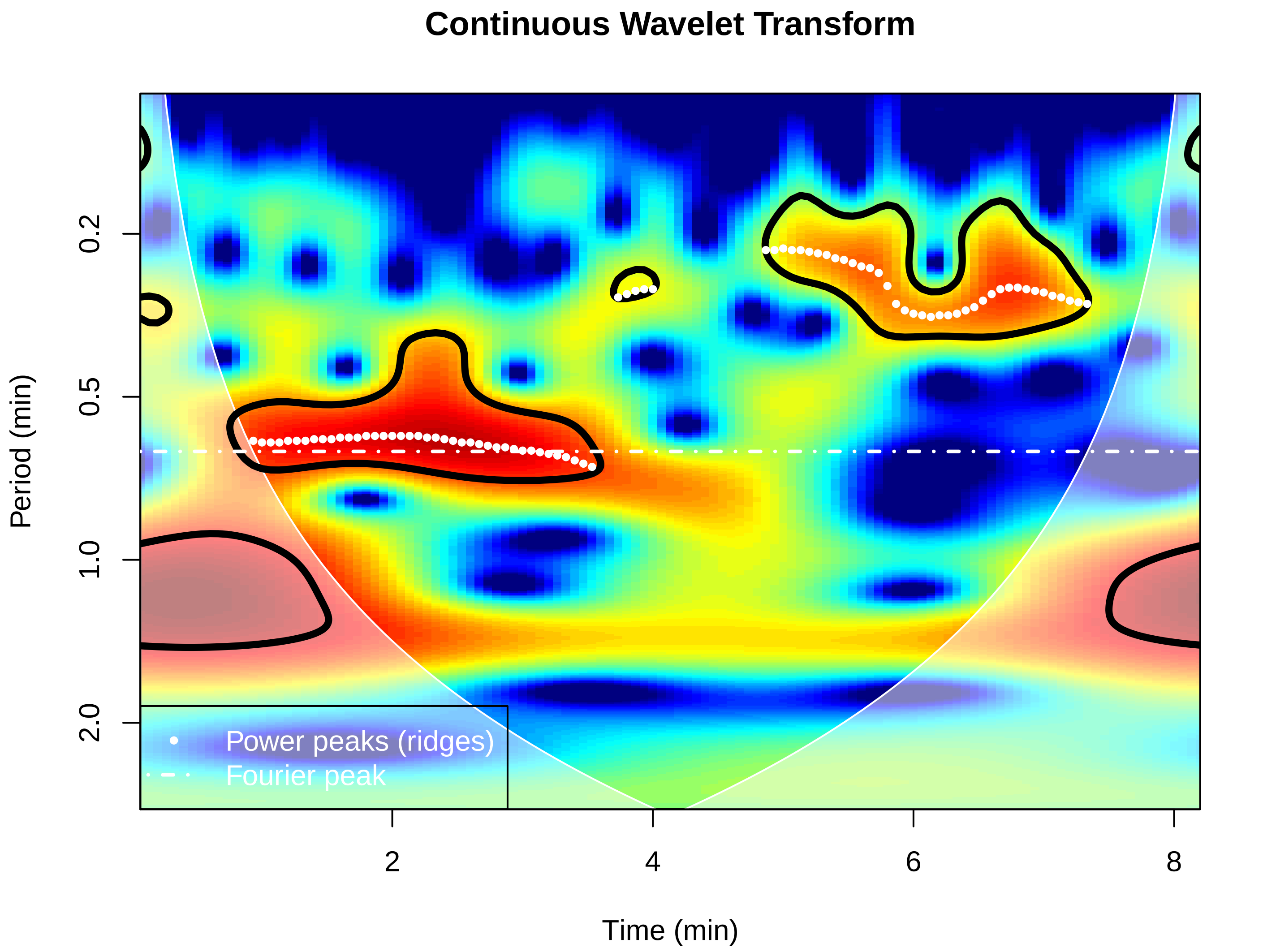
Continuous Wavelet Transform
Period (min)
0.2
0.5
1.0
2.0
2
4
6
8
Time (min)
Power peaks (ridges)
Fourier peak
