# Supplementary material for: Oscillatory signatures underlie growth regimes in Arabidopsis pollen tubes: computational methods to estimate tip location, periodicity, and synchronization in growing cells
Source: J Exp Bot. 2017 Mar 28;68(12):3267–81. doi: 10.1093/jxb/erx032 (PMC5853864; doi:10.1093/jxb/erx032)
Supplement: supplementary_dataset_S1 [file erx032_suppl_supplementary_dataset_s1.zip › CHUKNORRIS-master/out_exs/ratio_kymo/OscillationAnalysis_SHANK.FLUO_Arab_Col-0_Kymo_020813-A_CFP.txt.pdf]

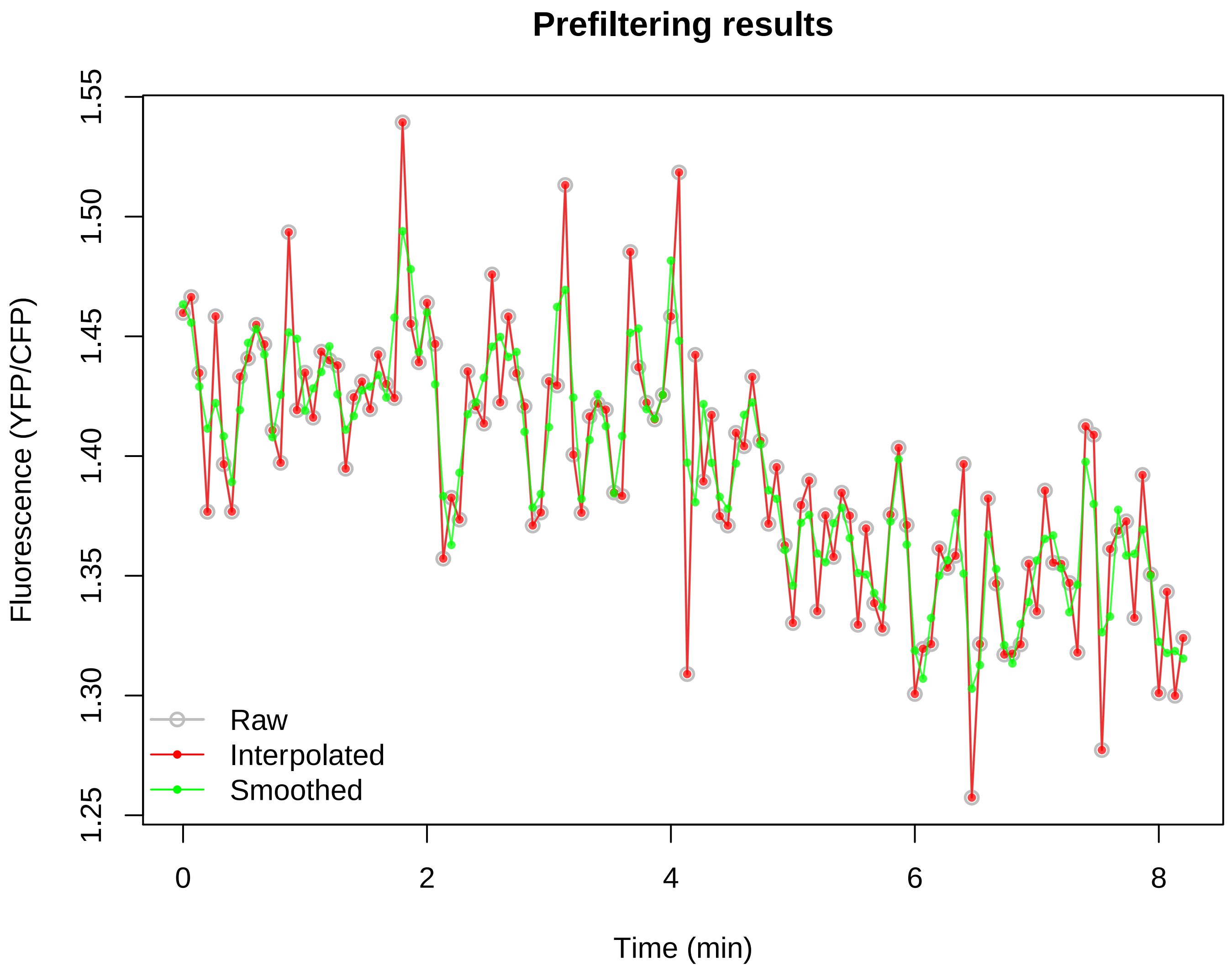

Prefiltering results
Fluorescence (YFP/CFP)
1.25
1.30
1.35
1.40
1.45
1.50
1.55
Time (min)
0
2
4
6
8
Raw
Interpolated
Smoothed

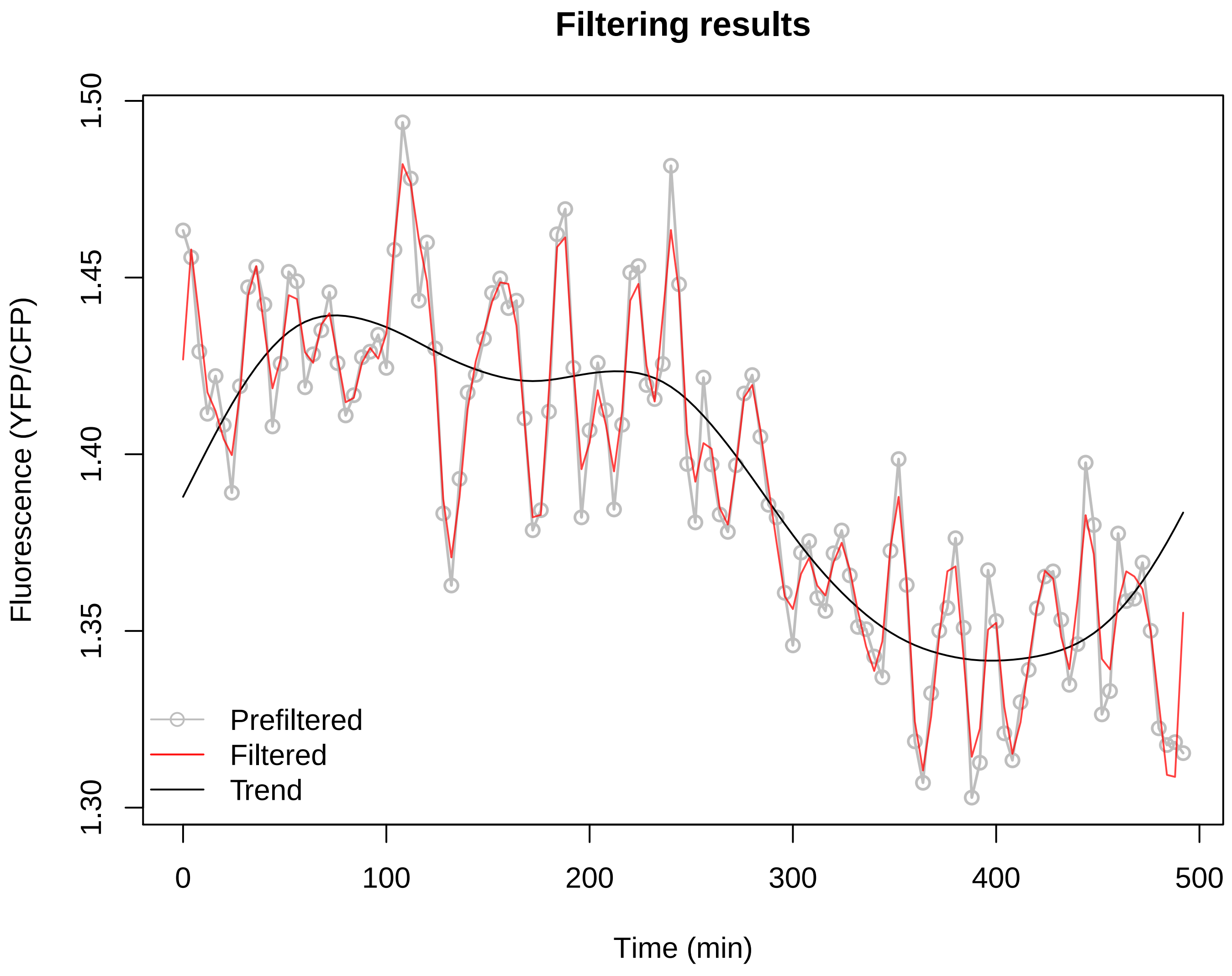

Filtering results
Fluorescence (YFP/CFP)
1.30
1.35
1.40
1.45
1.50
0
100
200
300
400
500
Time (min)
Prefiltered
Filtered
Trend

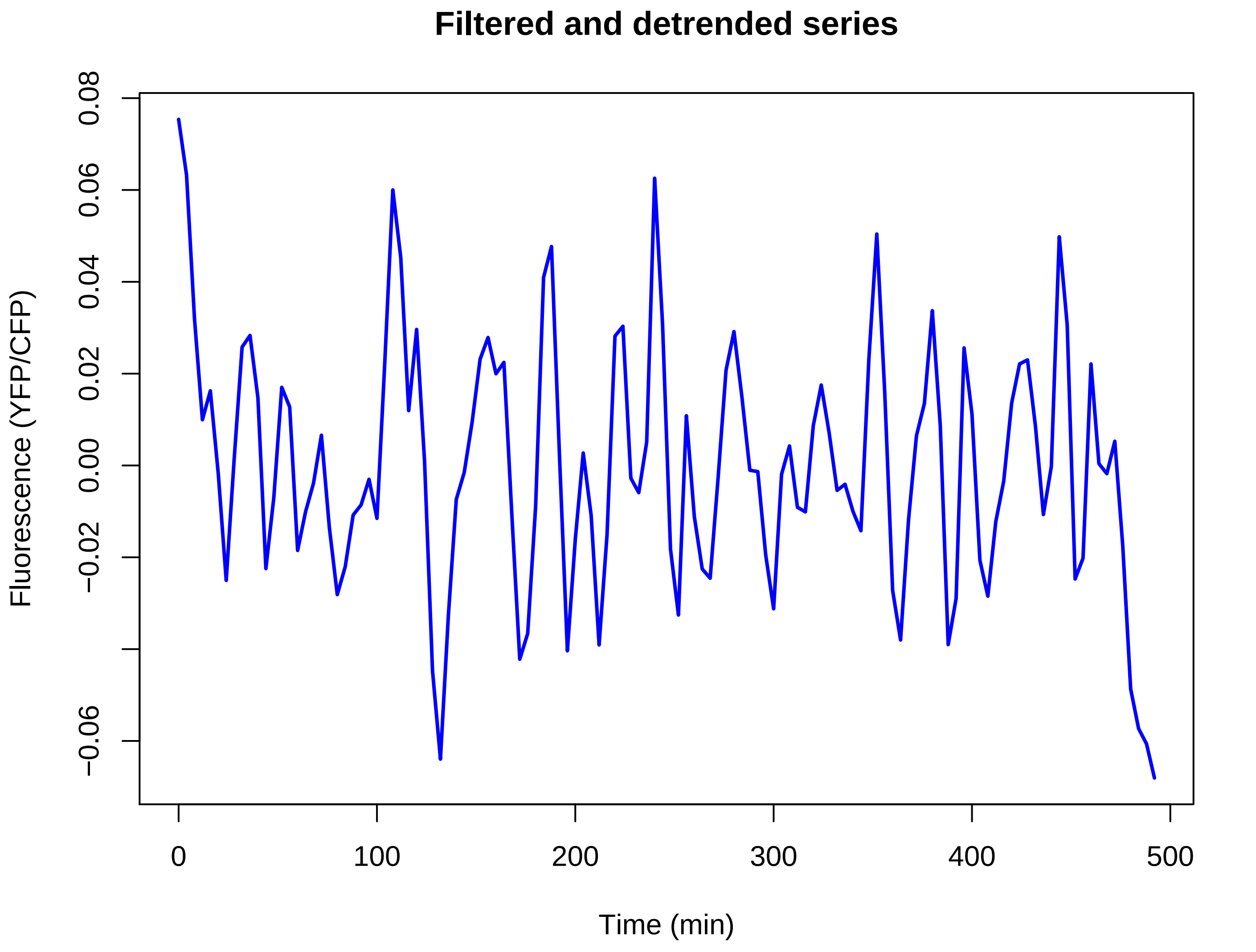

Filtered and detrended series
Fluorescence (YFP/CFP)
0.08
0.06
0.04
0.02
0.00
−0.02
−0.06
0
100
200
300
400
500
Time (min)

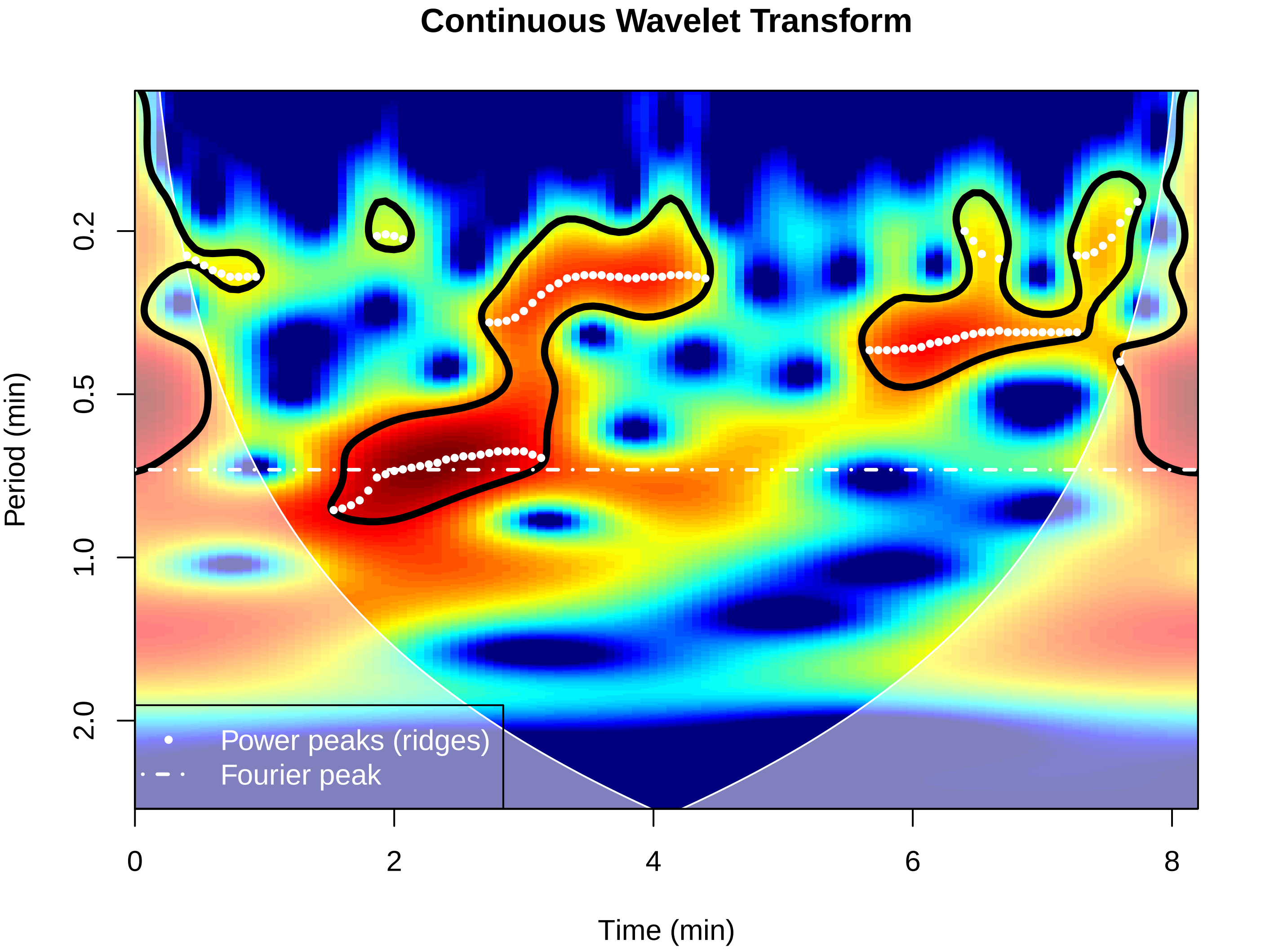

Continuous Wavelet Transform
Period (min)
0.2
0.5
1.0
2.0
Time (min)
0
2
4
6
8
Power peaks (ridges)
Fourier peak
