# Supplementary material for: Oscillatory signatures underlie growth regimes in Arabidopsis pollen tubes: computational methods to estimate tip location, periodicity, and synchronization in growing cells
Source: J Exp Bot. 2017 Mar 28;68(12):3267–81. doi: 10.1093/jxb/erx032 (PMC5853864; doi:10.1093/jxb/erx032)
Supplement: supplementary_dataset_S1 [file erx032_suppl_supplementary_dataset_s1.zip › CHUKNORRIS-master/out_exs/sync/OscillationAnalysis_TS_Object_1_Arab_Col-0_Track_190815-A.csv.pdf]

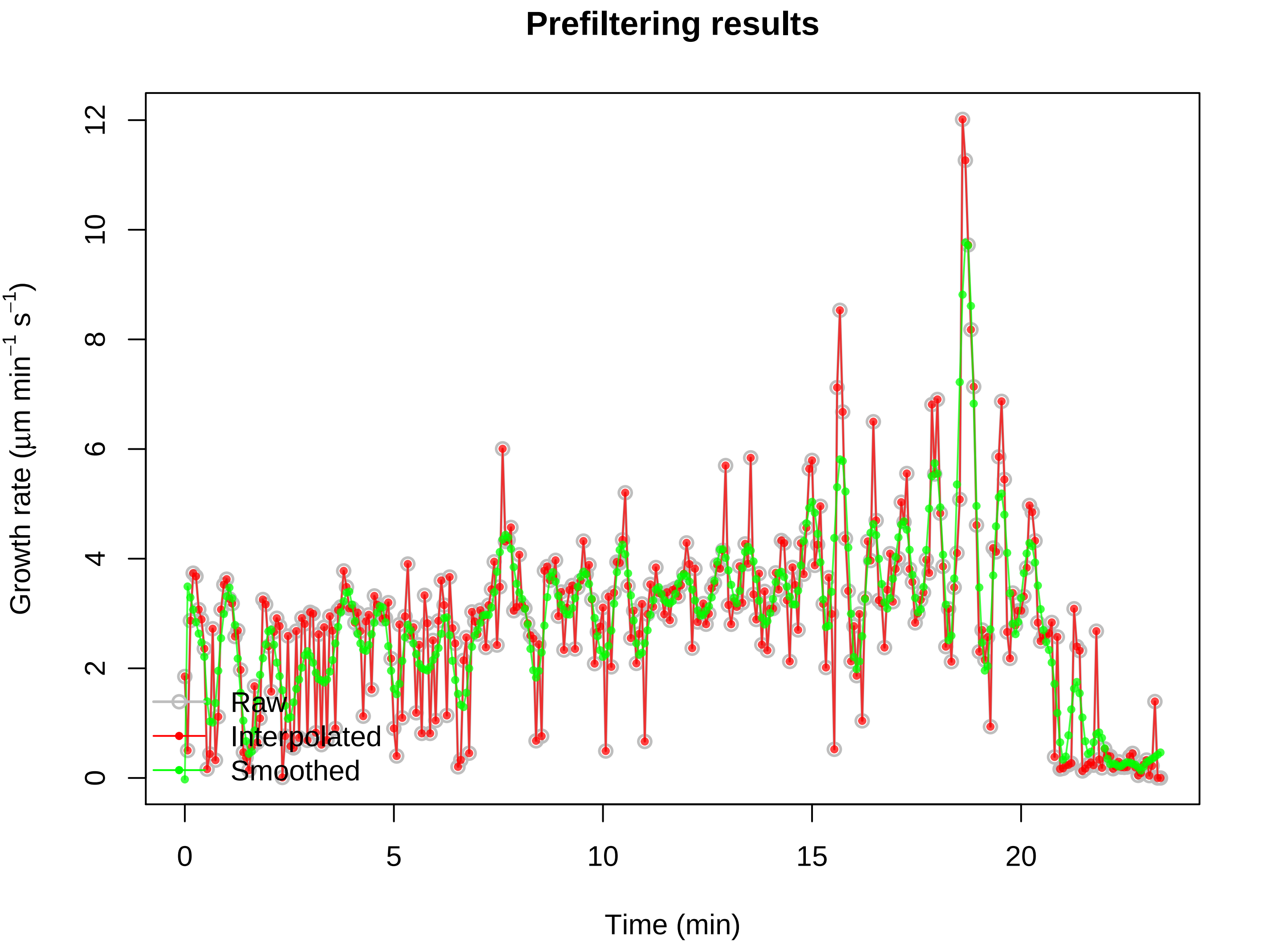
Prefiltering results
Growth rate (μm min⁻¹ s⁻¹)
Time (min)
0
2
4
6
8
10
12
0
5
10
15
20
Raw
Interpolated
Smoothed

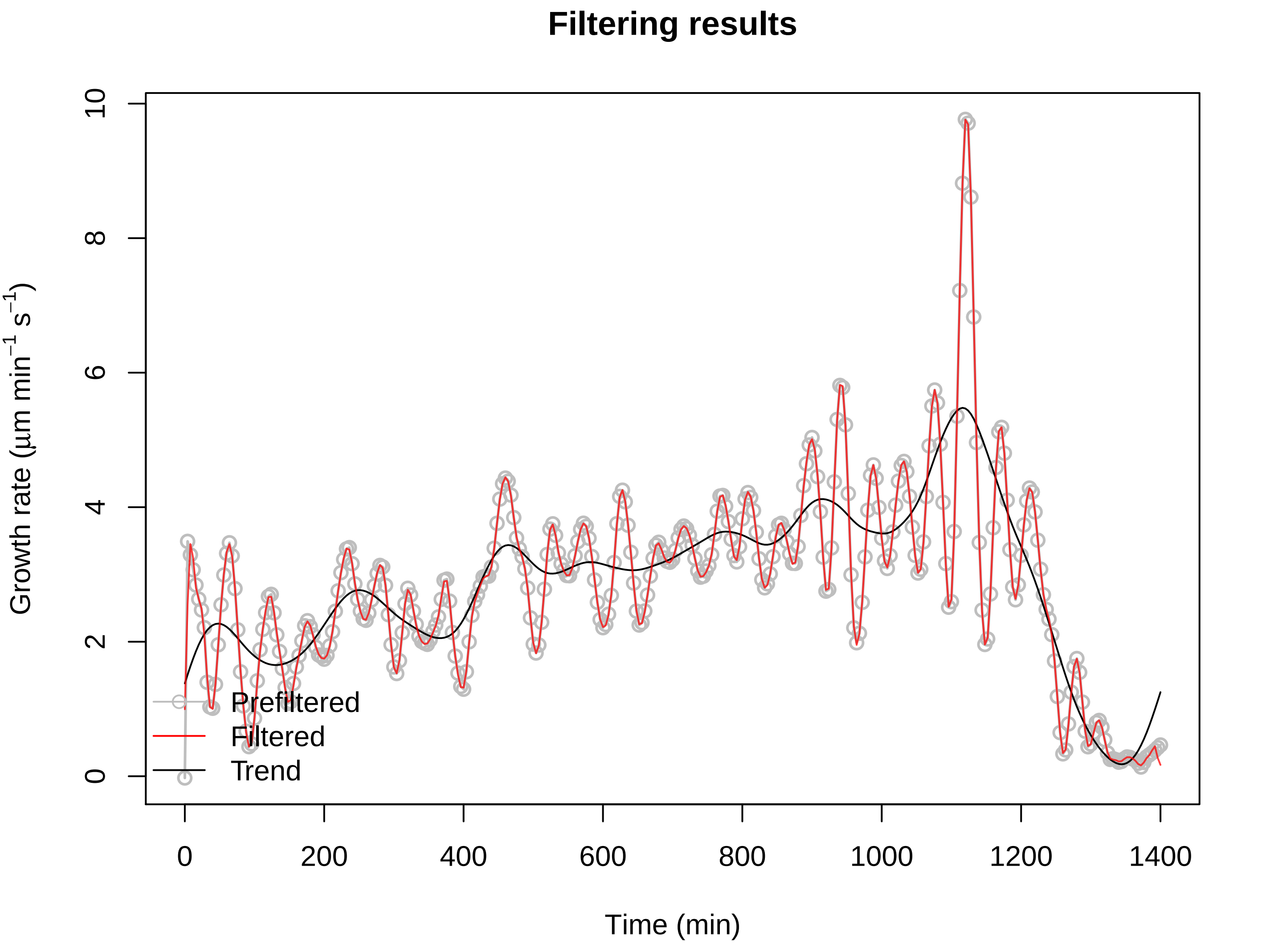
Filtering results
Growth rate (µm min⁻¹ s⁻¹)
Time (min)
0
2
4
6
8
10
0
200
400
600
800
1000
1200
1400
Prefiltered
Filtered
Trend

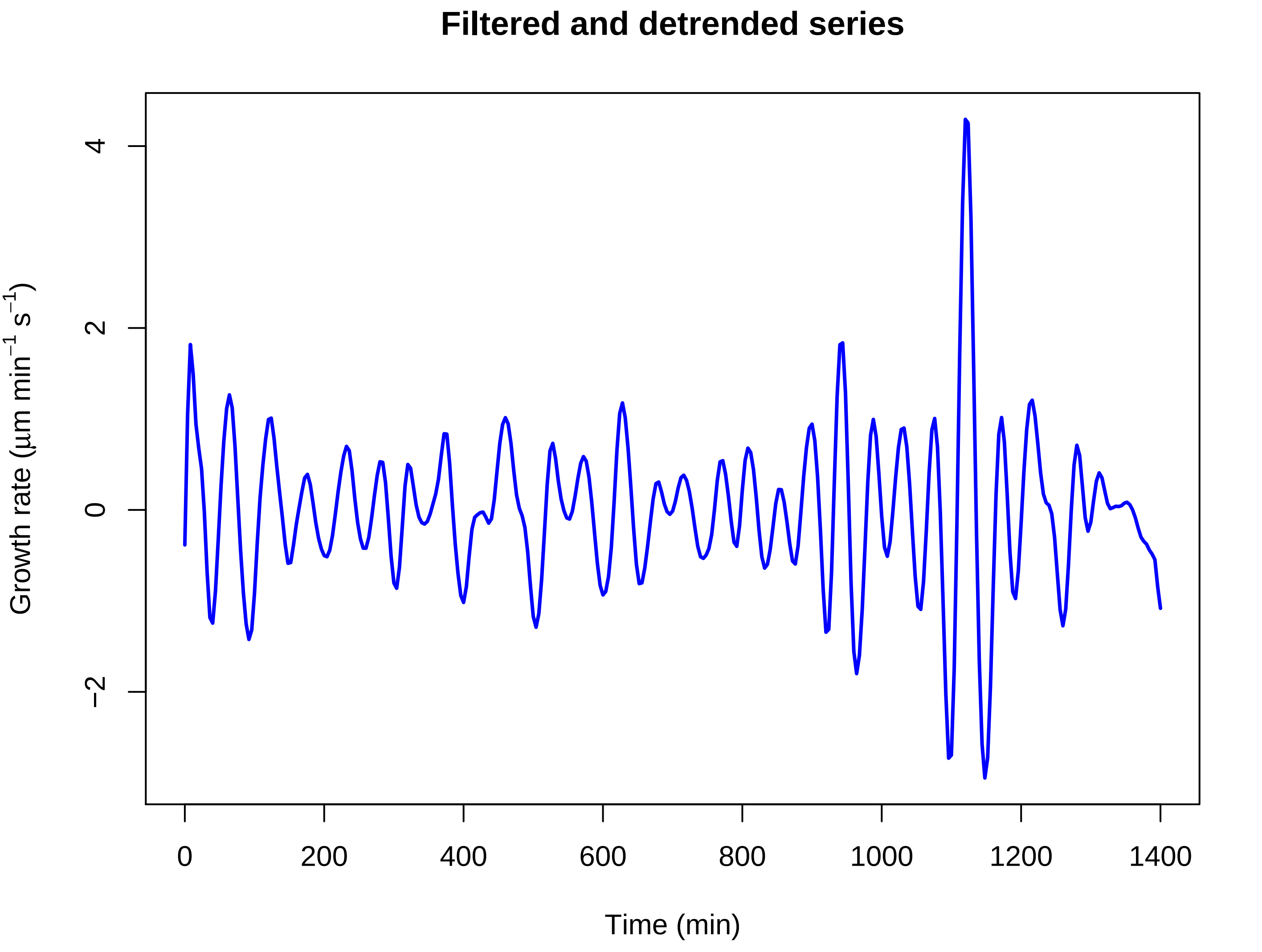

Filtered and detrended series
Growth rate (μm min−1 s−1)
4
2
0
−2
0
200
400
600
800
1000
1200
1400
Time (min)

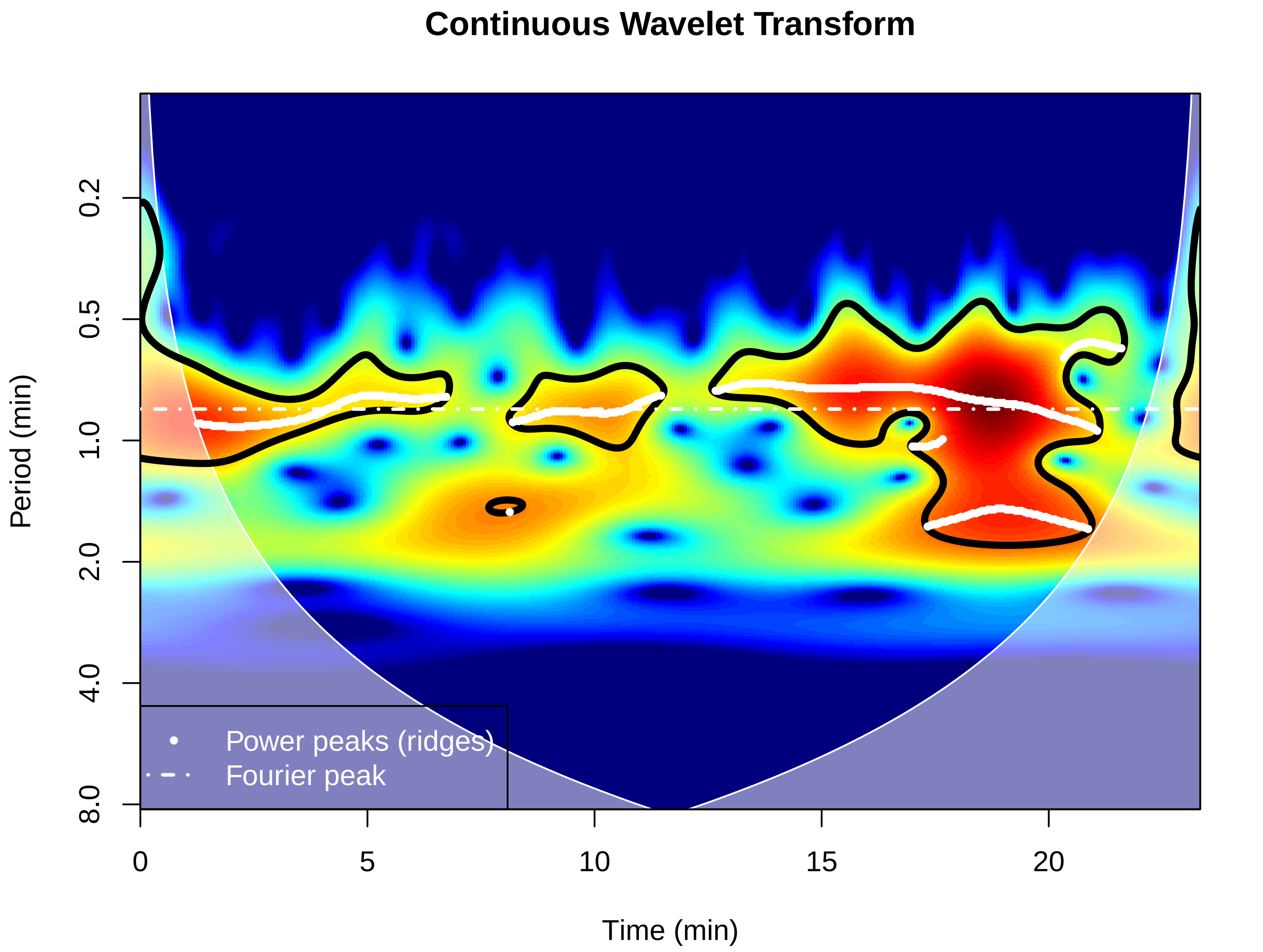
Continuous Wavelet Transform
Period (min)
0.2
0.5
1.0
2.0
4.0
8.0
Time (min)
0
5
10
15
20
Power peaks (ridges)
Fourier peak
