# Supplementary material for: Oscillatory signatures underlie growth regimes in Arabidopsis pollen tubes: computational methods to estimate tip location, periodicity, and synchronization in growing cells
Source: J Exp Bot. 2017 Mar 28;68(12):3267–81. doi: 10.1093/jxb/erx032 (PMC5853864; doi:10.1093/jxb/erx032)
Supplement: supplementary_dataset_S1 [file erx032_suppl_supplementary_dataset_s1.zip › CHUKNORRIS-master/out_exs/time_series/OscillationAnalysis_Arab_Col-0_H-VP_190815-A.csv.pdf]

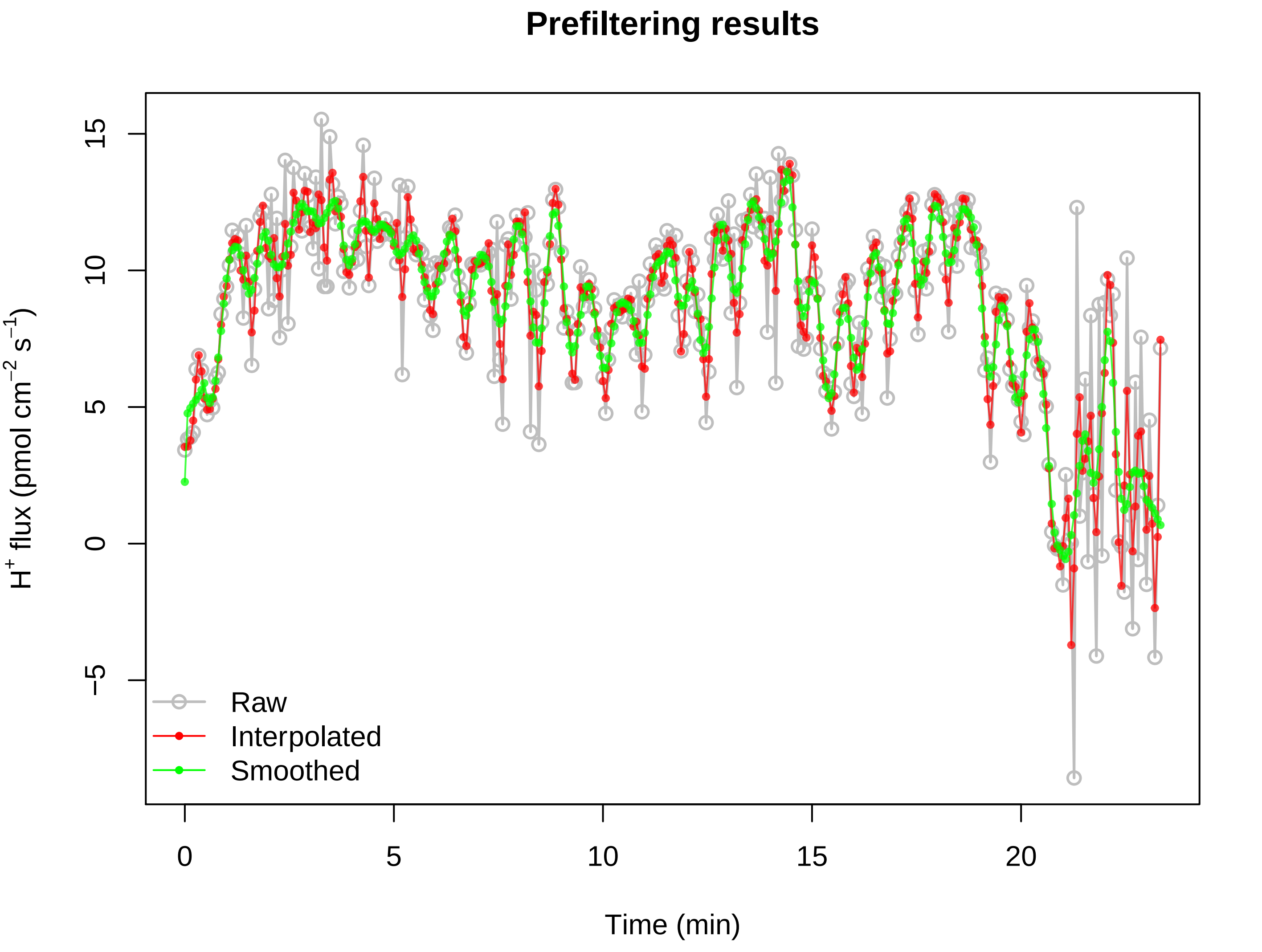

Prefiltering results
H+ flux (pmol cm−2 s−1)
Time (min)
15
10
5
0
−5
0
5
10
15
20
Raw
Interpolated
Smoothed

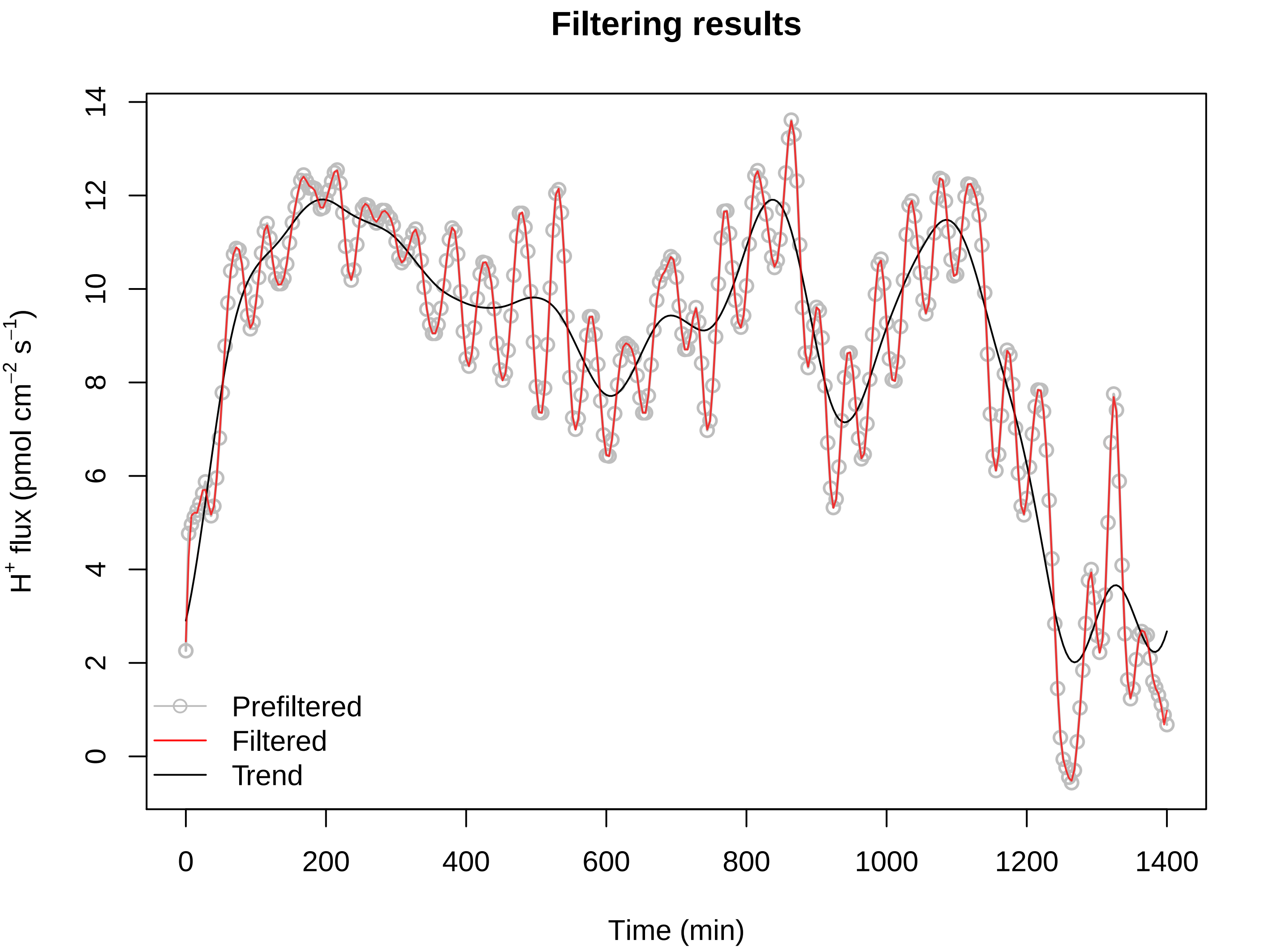

Filtering results
H+ flux (pmol cm−2 s−1)
Time (min)
0
200
400
600
800
1000
1200
1400
0
2
4
6
8
10
12
14
Prefiltered
Filtered
Trend

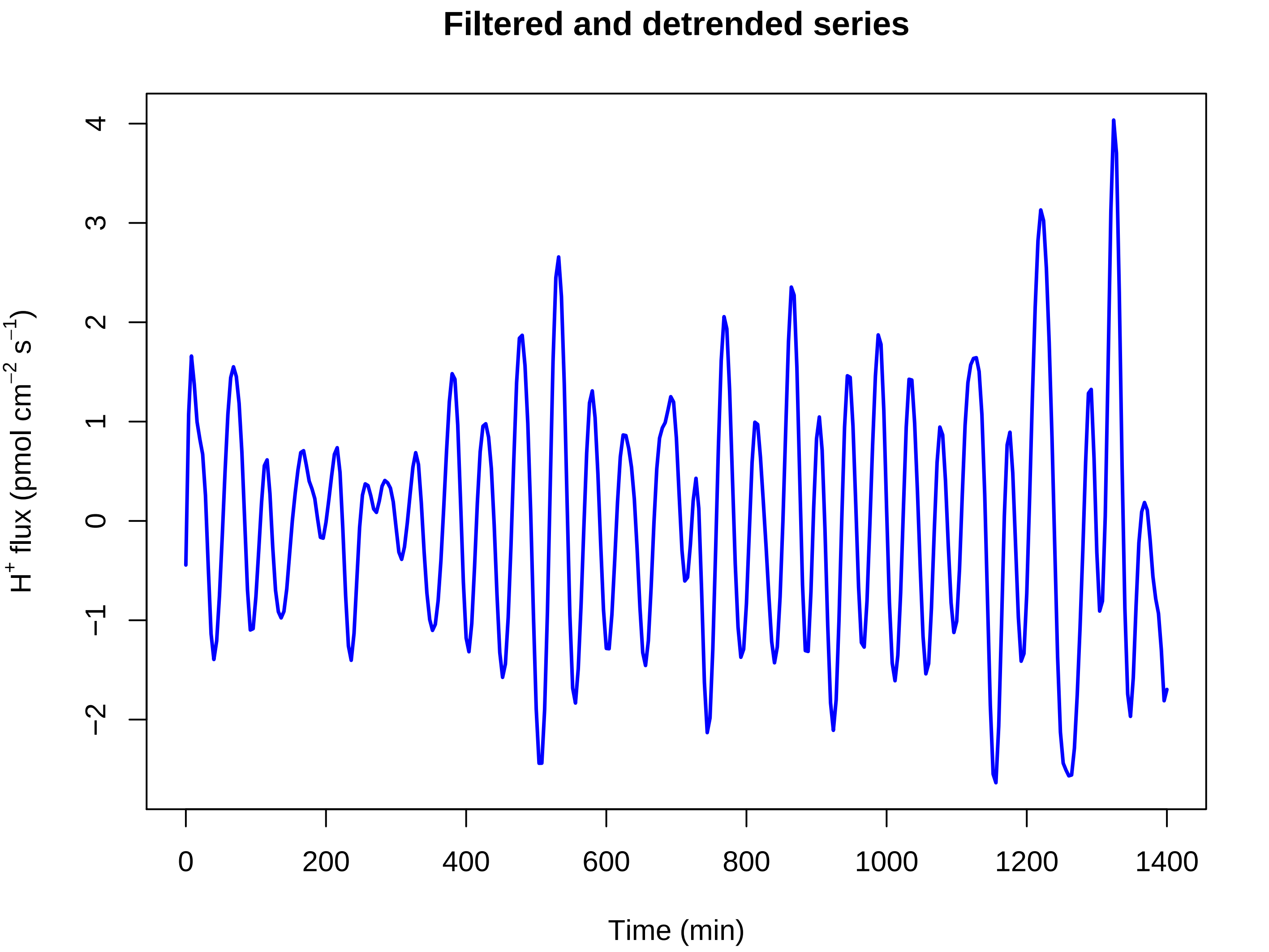
Filtered and detrended series
H^+ flux (pmol cm^{-2} s^{-1})
Time (min)
4
3
2
1
0
−1
−2
0
200
400
600
800
1000
1200
1400

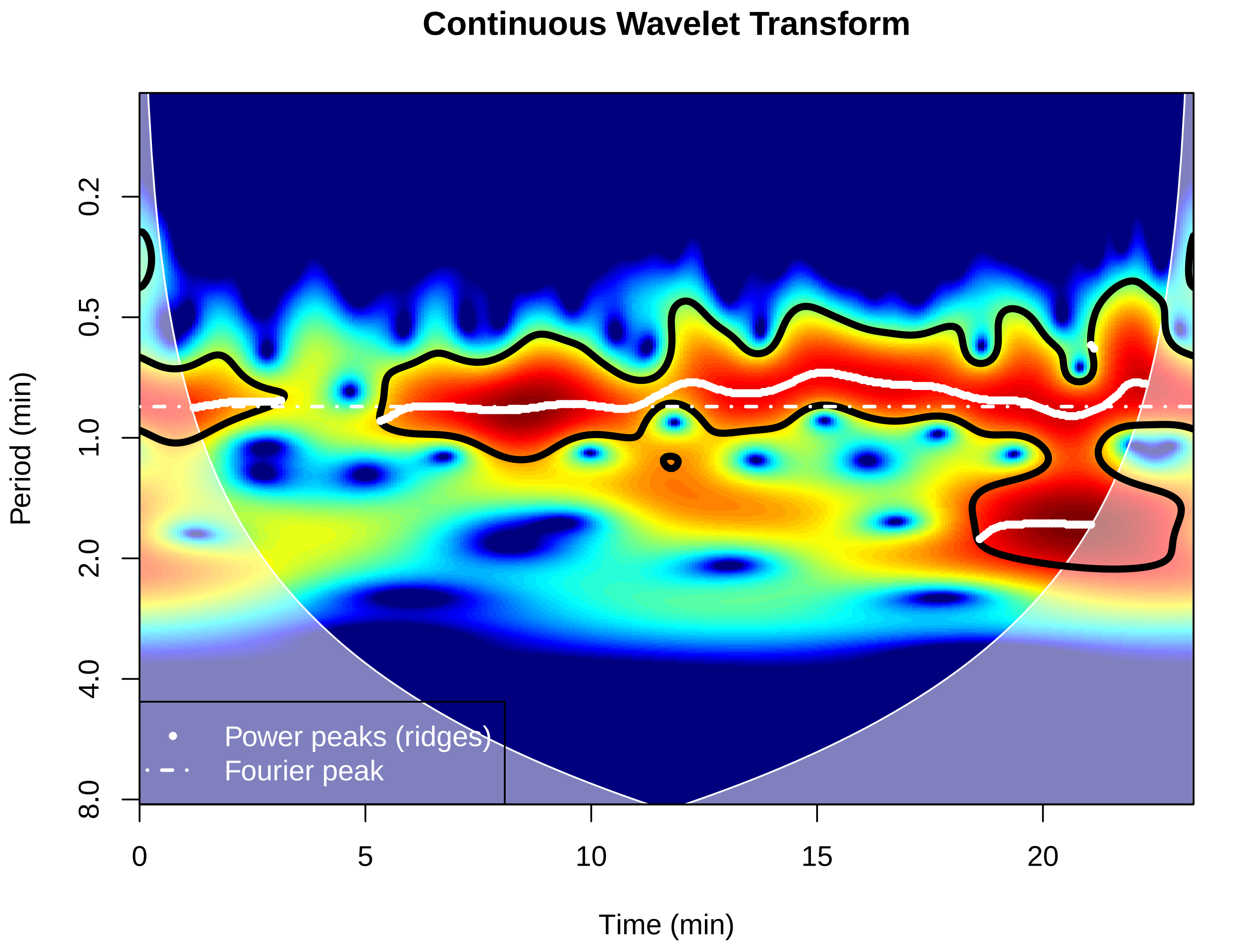

Continuous Wavelet Transform
Period (min)
0.2
0.5
1.0
2.0
4.0
8.0
Time (min)
0
5
10
15
20
Power peaks (ridges)
Fourier peak
