# Supplementary figures and images for: Oscillatory signatures underlie growth regimes in Arabidopsis pollen tubes: computational methods to estimate tip location, periodicity, and synchronization in growing cells
Source: J Exp Bot. 2017 Mar 28;68(12):3267–81. doi: 10.1093/jxb/erx032 (PMC5853864; doi:10.1093/jxb/erx032)

# YFP kymograph

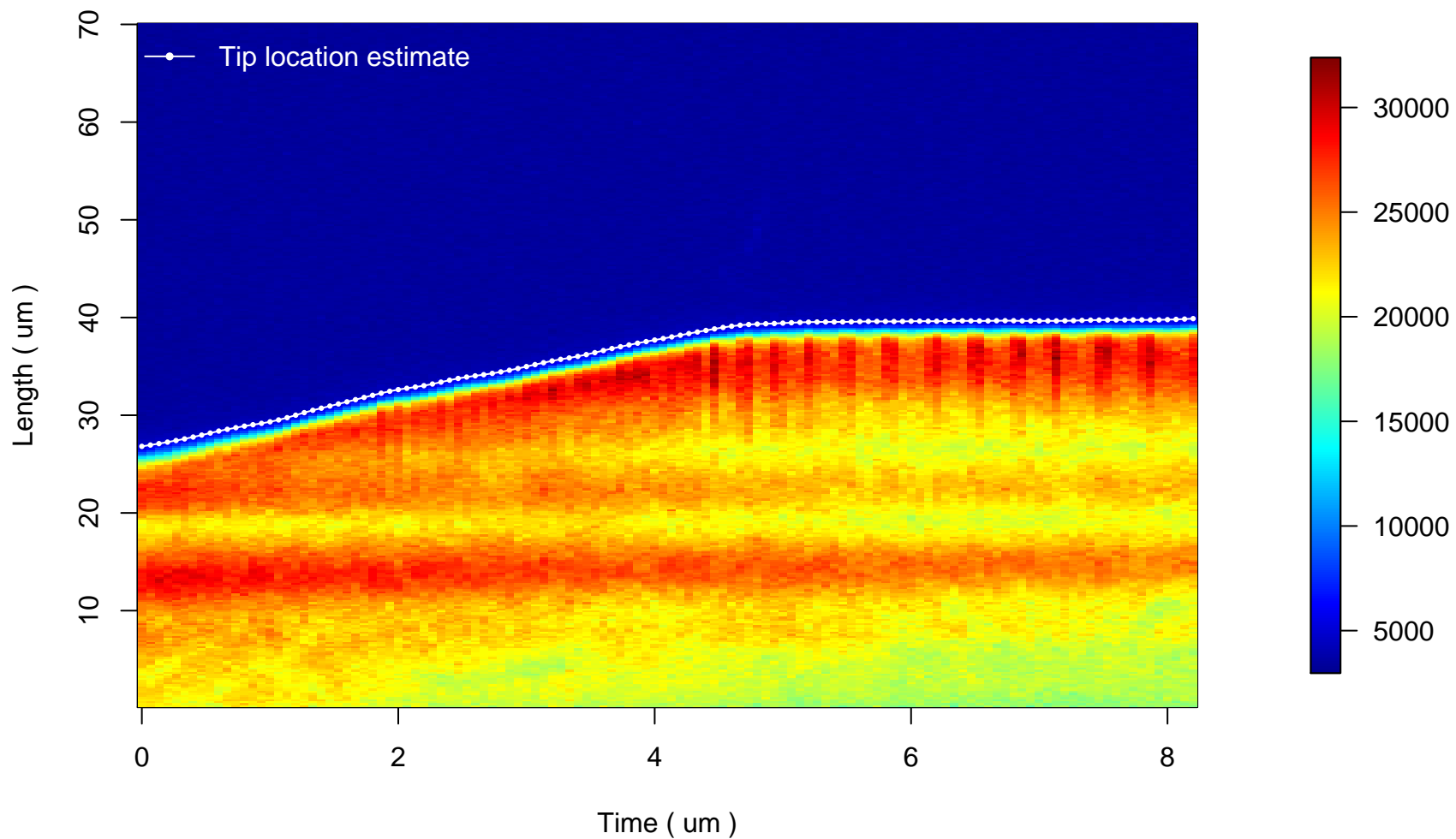

**Ratiometric tip aligned kymograph**

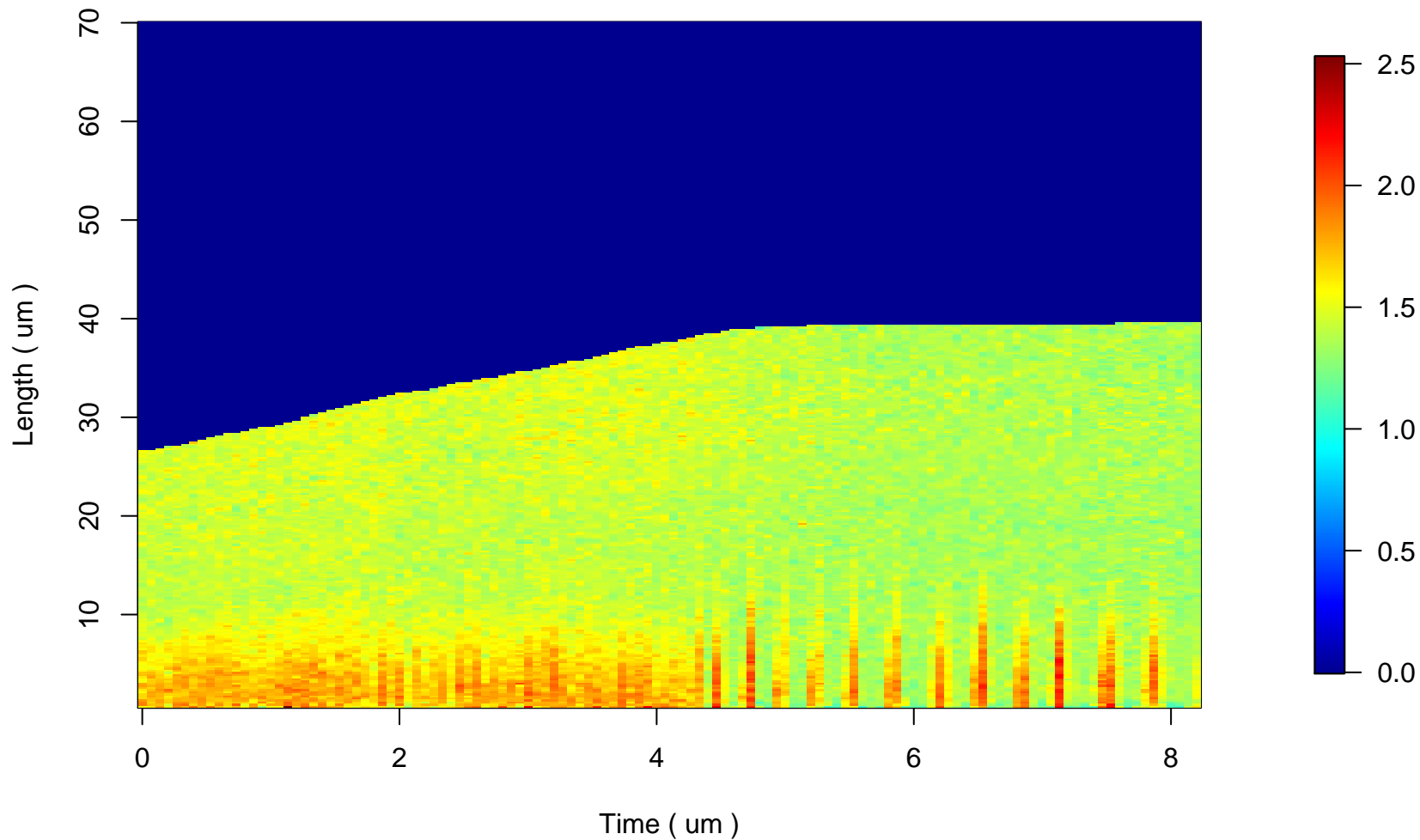

Supplement: supplementary_dataset_S1 [file erx032_suppl_supplementary_dataset_s1.zip › CHUKNORRIS-master/out_exs/ratio_kymo/RatioKymo_Arab_Col-0_Kymo_020813-A_CFP.txt.pdf]

## Filtered and matched series

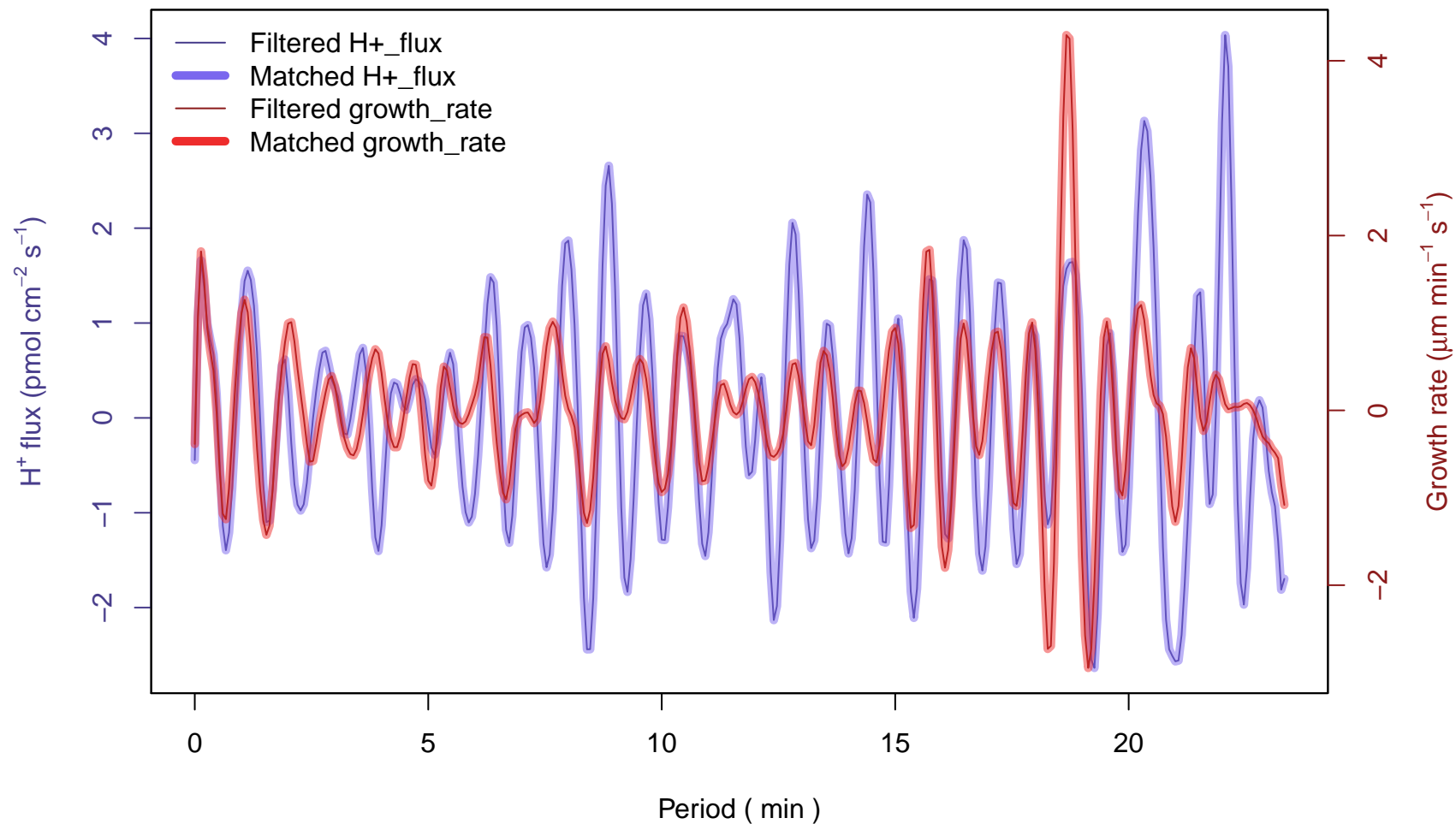

# Cross Wavelet Transform

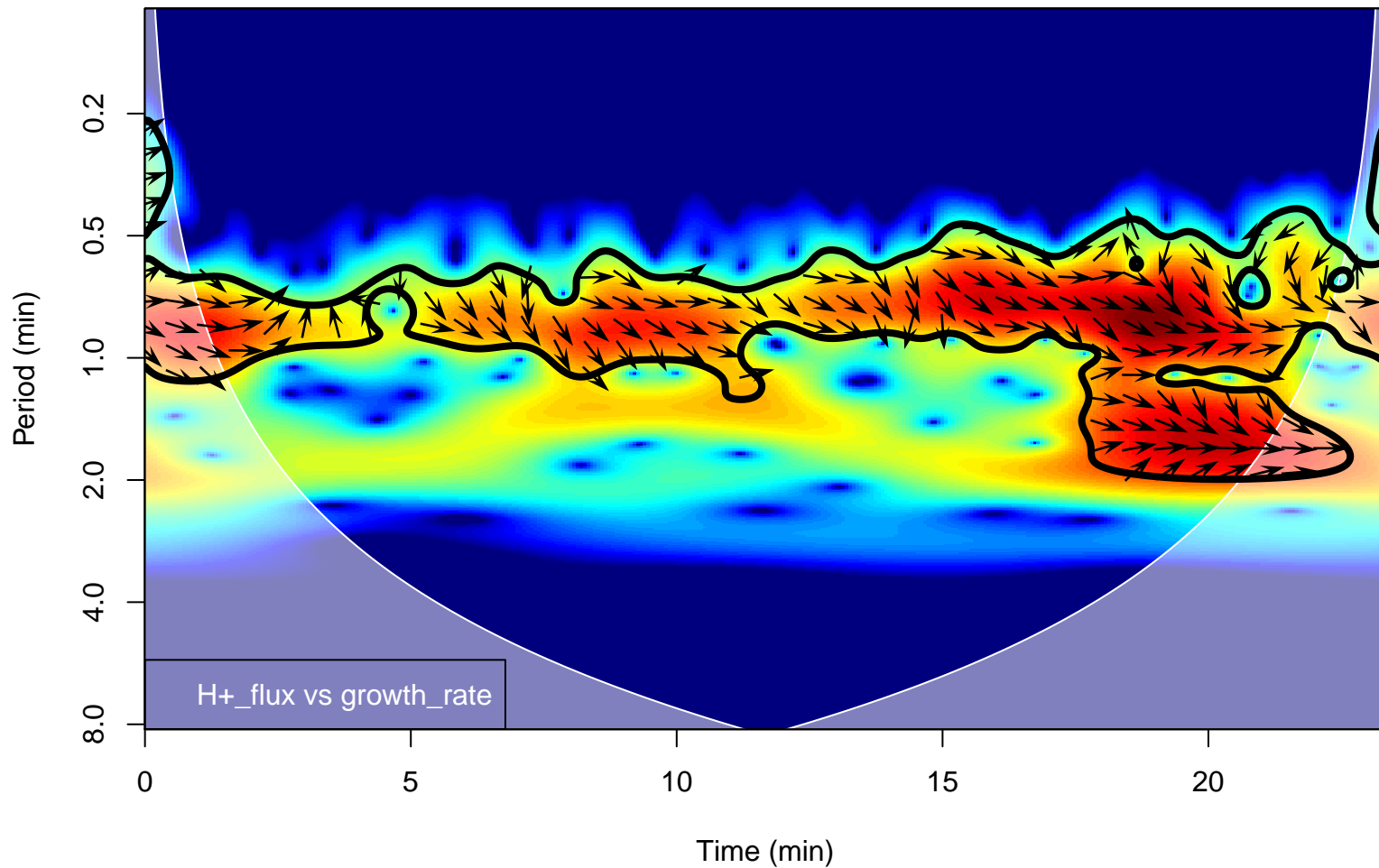

Supplement: supplementary_dataset_S1 [file erx032_suppl_supplementary_dataset_s1.zip › CHUKNORRIS-master/out_exs/sync/Sync_Arab_Col-0_H-VP_190815-A.csv_VS_TS_Object_1_Arab_Col-0_Track_190815-A.csv.pdf]
