# Supplementary material for: Oscillatory signatures underlie growth regimes in Arabidopsis pollen tubes: computational methods to estimate tip location, periodicity, and synchronization in growing cells
Source: J Exp Bot. 2017 Mar 28;68(12):3267–81. doi: 10.1093/jxb/erx032 (PMC5853864; doi:10.1093/jxb/erx032)
Supplement: supplementary_dataset_S1 [file Kymo_Arab_Col-0_Kymo_190815-A.txt.pdf › CHUKNORRIS-master/out_exs/kymo/Kymo_Arab_Col-0_Kymo_190815-A.txt.pdf]

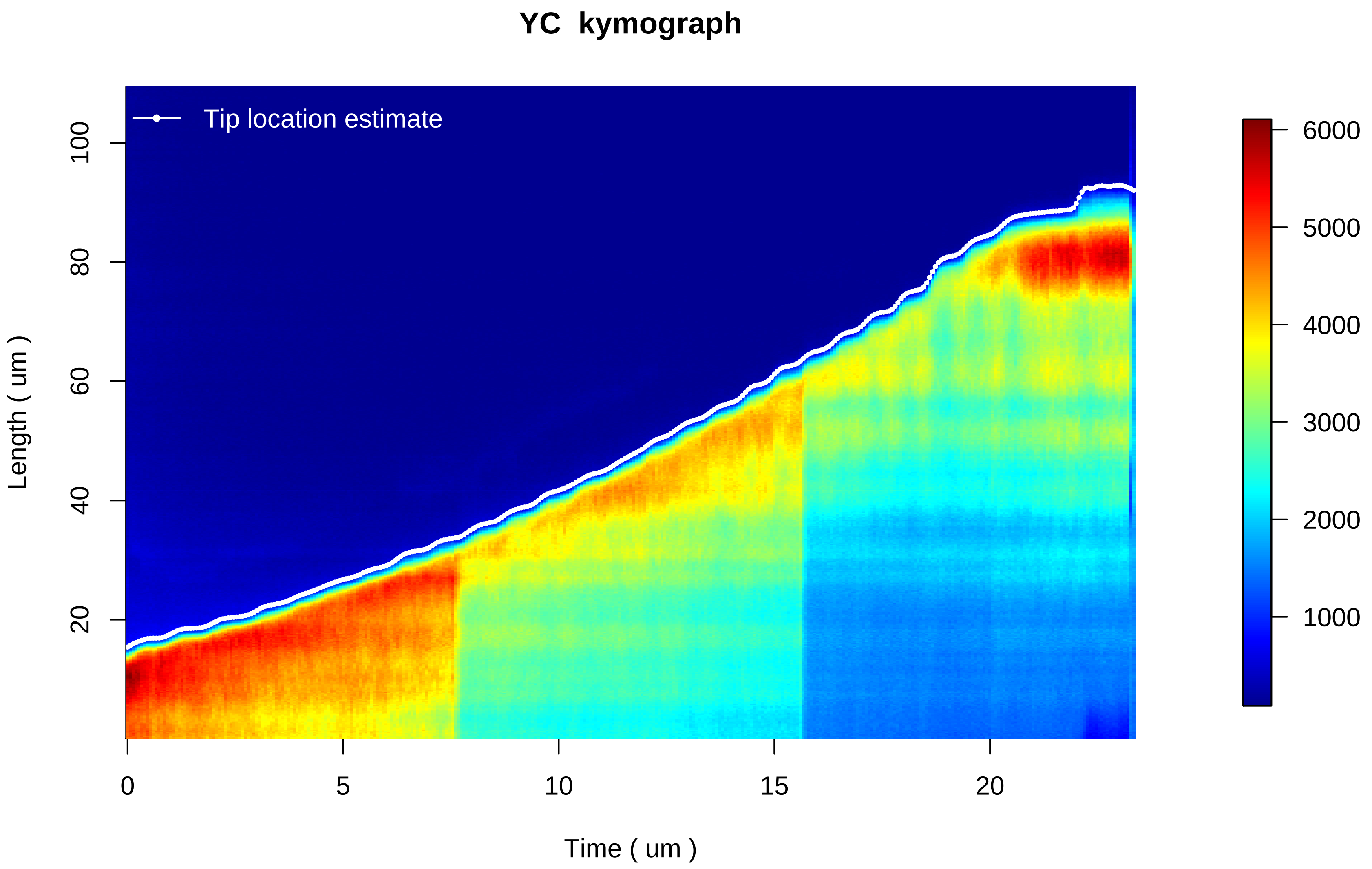
YC kymograph
Tip location estimate
Length (um)
Time (um)
0
5
10
15
20
20
40
60
80
100
1000
2000
3000
4000
5000
6000
