# Supplementary material for: Oscillatory signatures underlie growth regimes in Arabidopsis pollen tubes: computational methods to estimate tip location, periodicity, and synchronization in growing cells
Source: J Exp Bot. 2017 Mar 28;68(12):3267–81. doi: 10.1093/jxb/erx032 (PMC5853864; doi:10.1093/jxb/erx032)
Supplement: supplementary_Table_S1_Figures_S1_S5 [file erx032_suppl_supplementary_table_s1_figures_s1_s5.pdf]

Oscillatory signatures underlie growth regimes in Arabidopsis pollen tubes: computational methods to estimate tip location, periodicity and synchronization in growing cells

Daniel S. C. Damineli, Maria Teresa Portes, José A. Feijó

SUPPLEMENTARY DATA

First the supplementary figures are presented Fig S1-S5, followed by the supplementary Table S1, and legends for the remaining files (Data S1 and Video S1 and Video S2).

Figures

Figure S1A

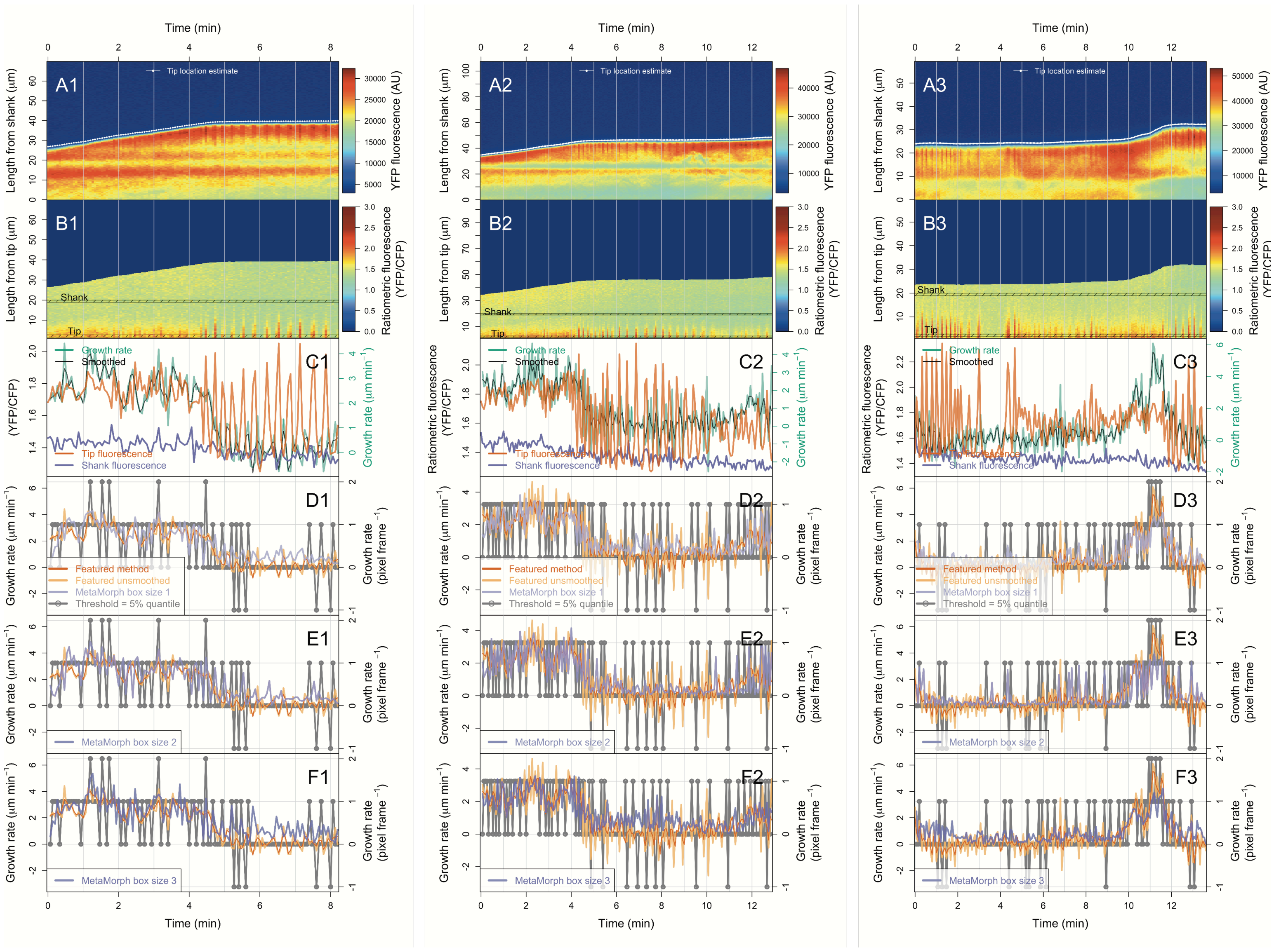

Figure S1B

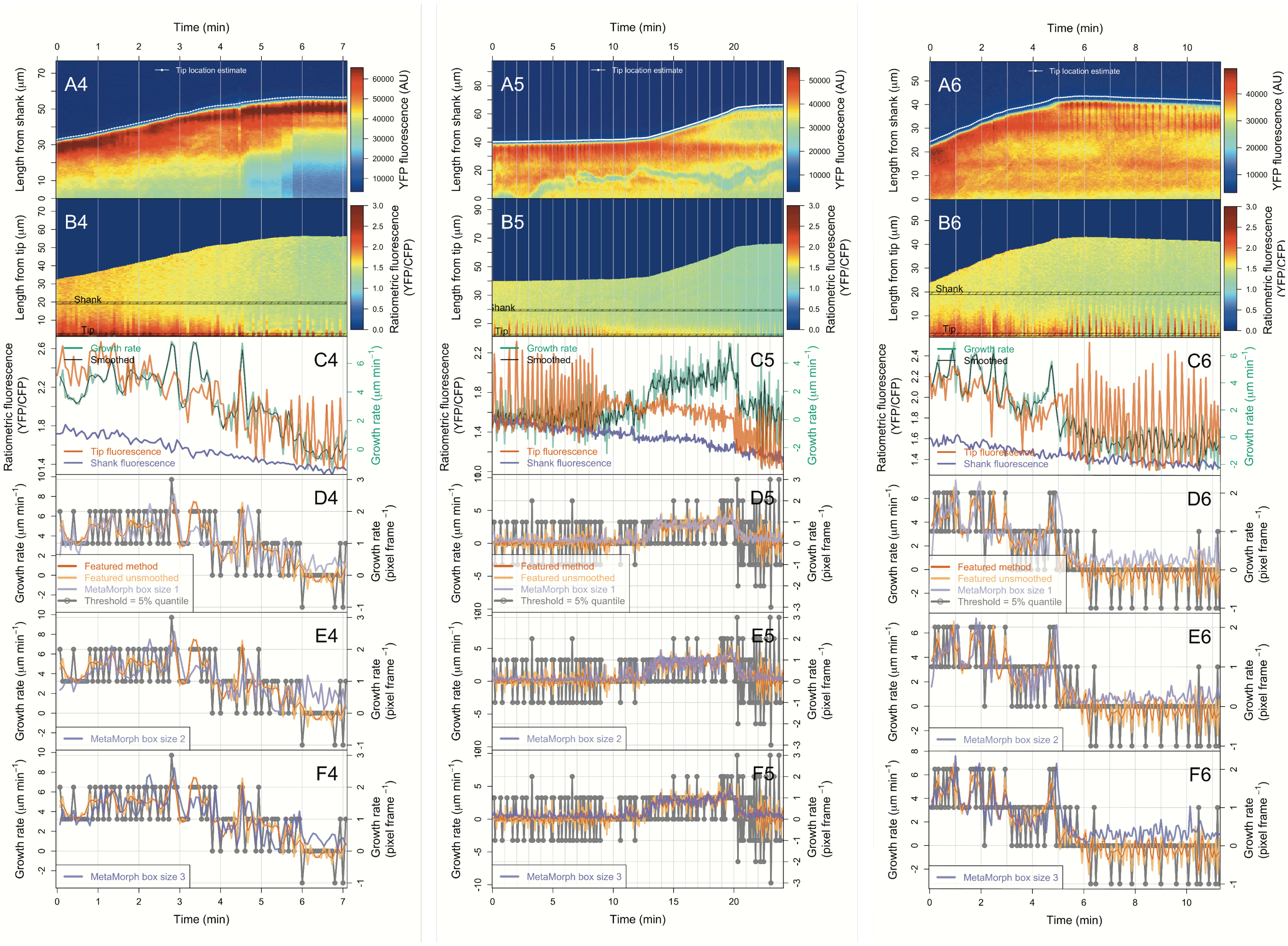

Fig. S1A-B: Individual ratiometric kymographs used in this work. For all six series (i=1,…,6), panel A_i shows the YFP channel, B_i the tip aligned kymograph using the featured detection method, C_i all three time series extracted, being growth rate, tip and shank fluorescence, D_i the comparison of growth rate series estimated by the MetaMorph algorithm using box size with the threshold and featured method, E_i the same as the previous but with MetaMorph box size 2 and F_i with MetaMorph box size 3.

Figure S2

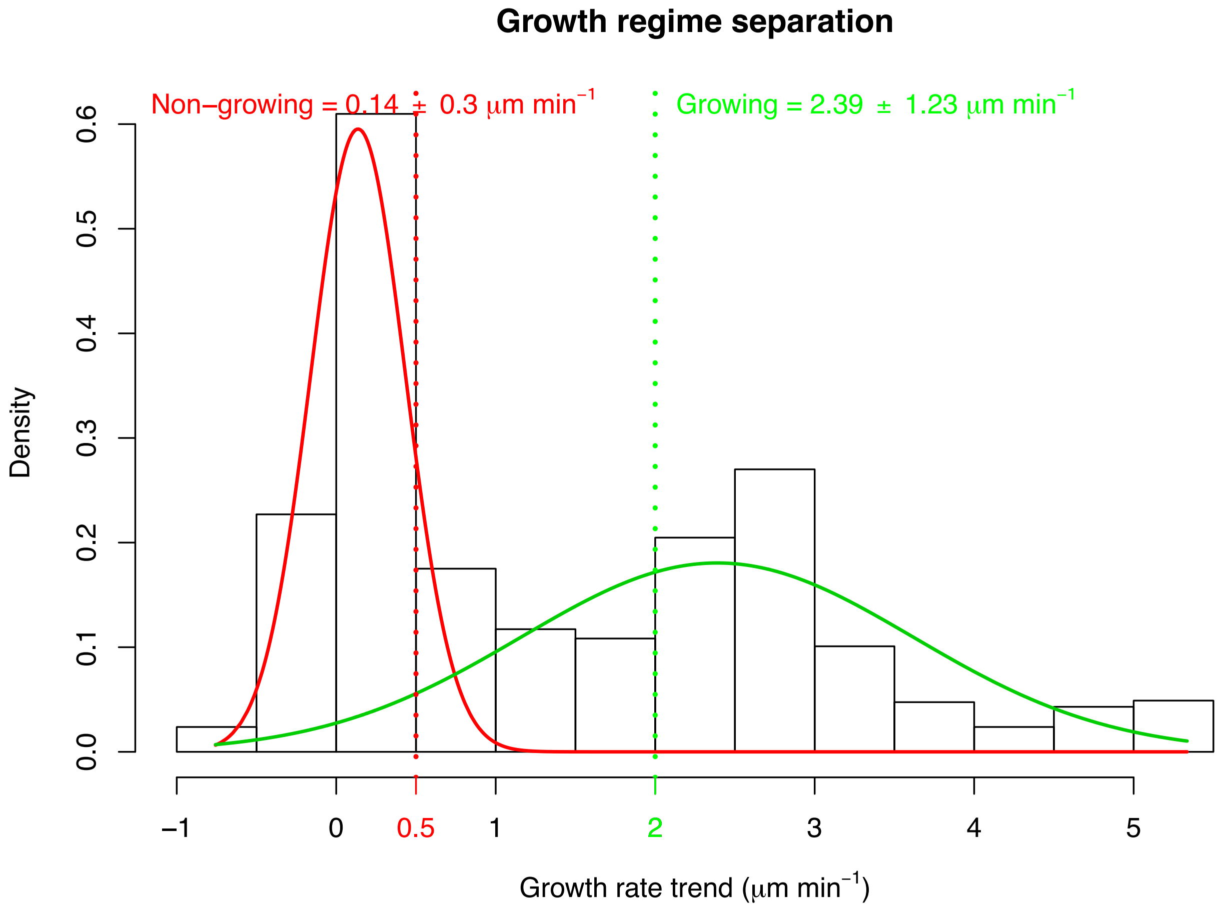

Fig. S2: Distribution of average growth rates. Distribution of average growth rates obtained from extracting the trend of all points of the 6 ratiometric kymographs analyzed here. A 2-component Gaussian Mixture model was used to estimate the mean and standard deviation of two underlying populations as to justify the classification of regimes into "non-growing" and "growing". Dotted lines show arbitrary values adopted that provide a good separation of the distributions, although changes in these thresholds had little effect on the comparisons made using these categories.

Figure S3

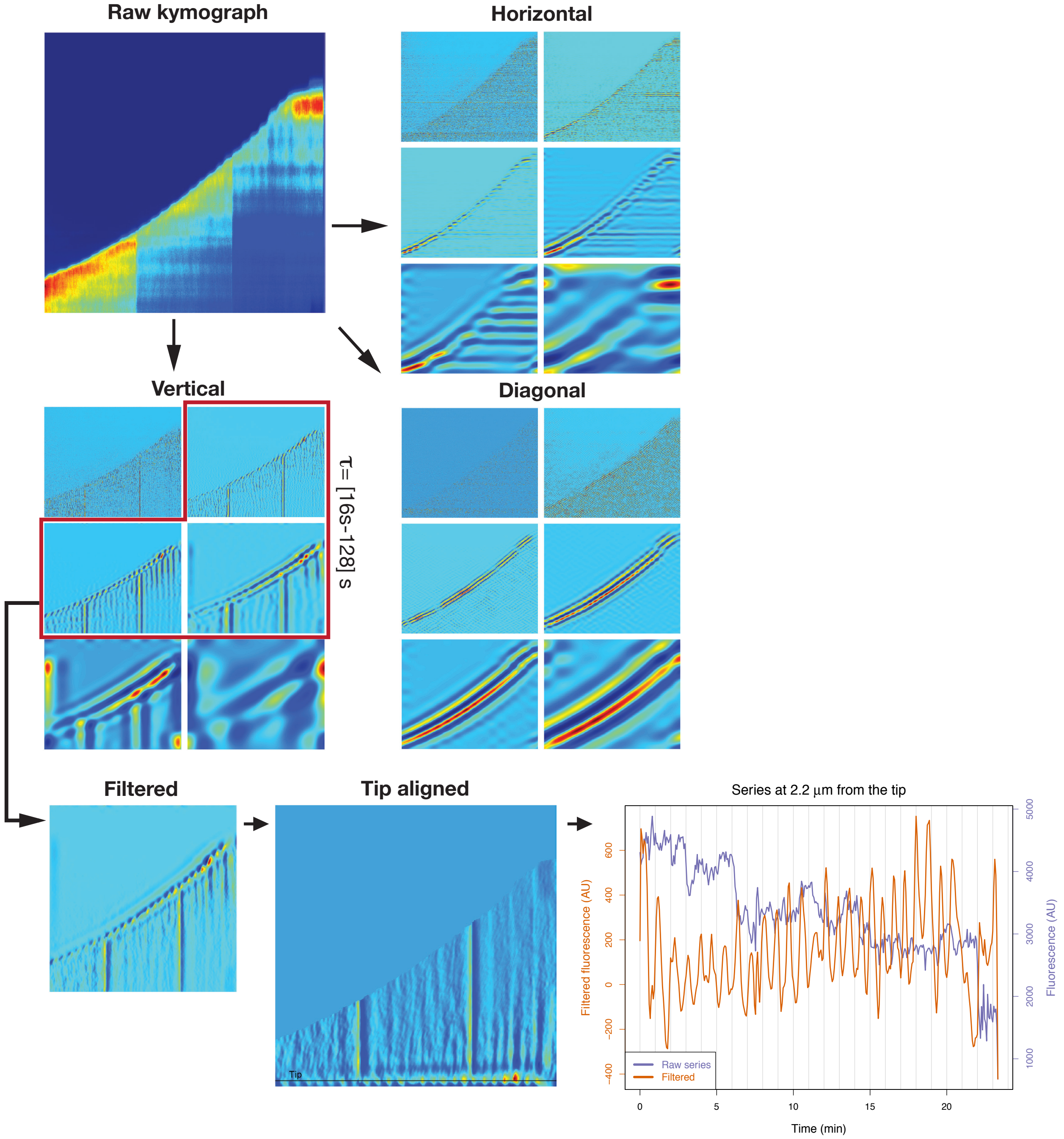

Fig. S3: Single channel kymograph analysis module featuring the filtering procedure with an illustrative example.

Figure S4

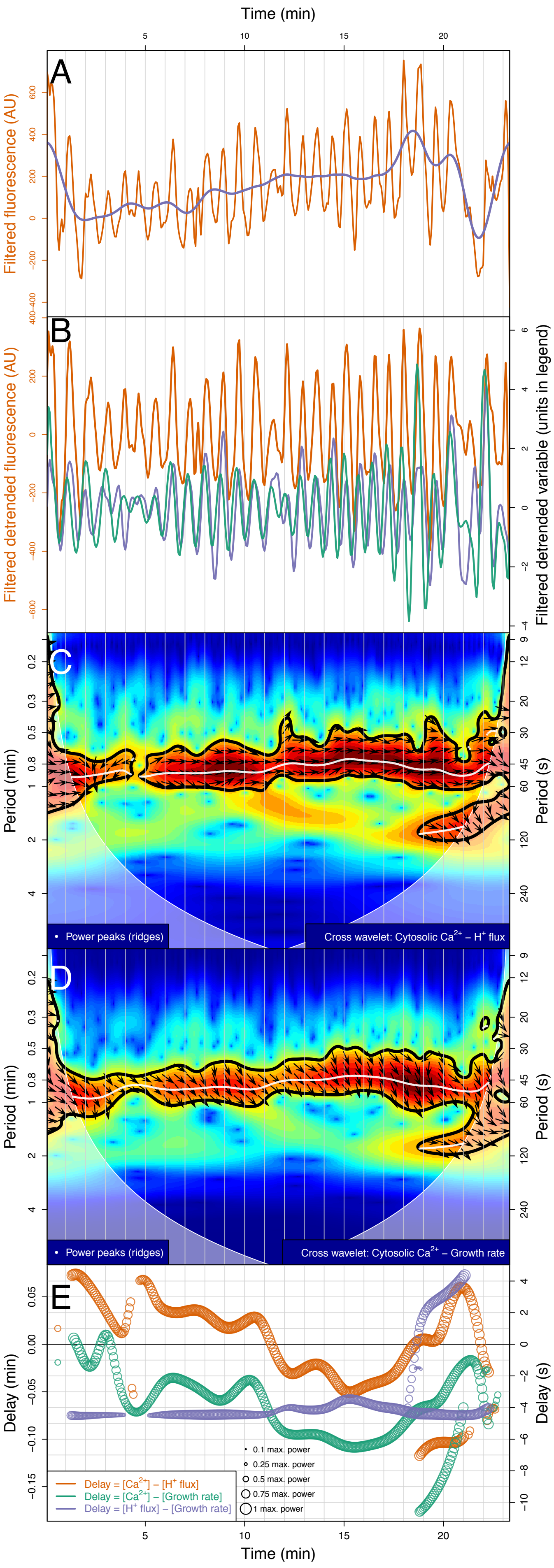
Time (min)
A
Filtered fluorescence (AU)
B
Filtered detrended fluorescence (AU)
Filtered detrended variable (units in legend)
C
Period (min)
Period (s)
Power peaks (ridges)
Cross wavelet: Cytosolic Ca2+ – H+ flux
D
Period (min)
Period (s)
Power peaks (ridges)
Cross wavelet: Cytosolic Ca2+ – Growth rate
E
Delay (min)
Delay (s)
0.1 max. power
0.25 max. power
0.5 max. power
0.75 max. power
1 max. power
Delay = [Ca2+] – [H+ flux]
Delay = [Ca2+] – [Growth rate]
Delay = [H+ flux] – [Growth rate]
Time (min)

Fig. S4: Oscillations in cytosolic Ca^{2+} extracted from the single channel kymograph showing synchronization with H^+ flux and growth rate oscillations. A – Ca^{2+} trace obtained using the 2D filter described for single channel kymographs still showing some trend to be removed. B – All 3 detrended and smoothed series Ca^{2+}, H^+ flux and growth rate showing joint oscillations. C – Cross wavelet transform of Ca^{2+} and H^+ flux series, showing regions of significant joint periodicity with arrows indicating the phase relationship. The arrows point to the direction of the trigonometric circle, corresponding to the local phase angle between two series (e.g. *x* and *y*). Both series are in phase if arrows point to the right ($\Delta\varphi = 0$), in anti-phase if pointing to the left ($\Delta\varphi = \pi$), *x* phase leads *y* if pointing up (i.e. *x* occurs before; $\Delta\varphi = \frac{\pi}{2}$), while *y* phase leads *x* if pointing down (i.e. *x* occurs after; $\Delta\varphi = \frac{3\pi}{2}$). Colors correspond to the power of specific components through time (time-frequency space) with significant ($p<0.05$) periods circled in black. White dots indicate the peaks of the power spectrum detected (wavelet ridges), while the shaded regions correspond to the "cone of influence", a region in which analysis is not reliable. D – Cross wavelet transform of Ca^{2+} and growth rate series (details same as above). E – Time delay estimated with the cross wavelet analysis for all 3 pairs: Ca^{2+} and H^+ flux series; Ca^{2+} and growth rate; H^+ flux and growth rate oscillations. The size of the circles shows the relative oscillation amplitude (normalized by maximum power).

Figure S5

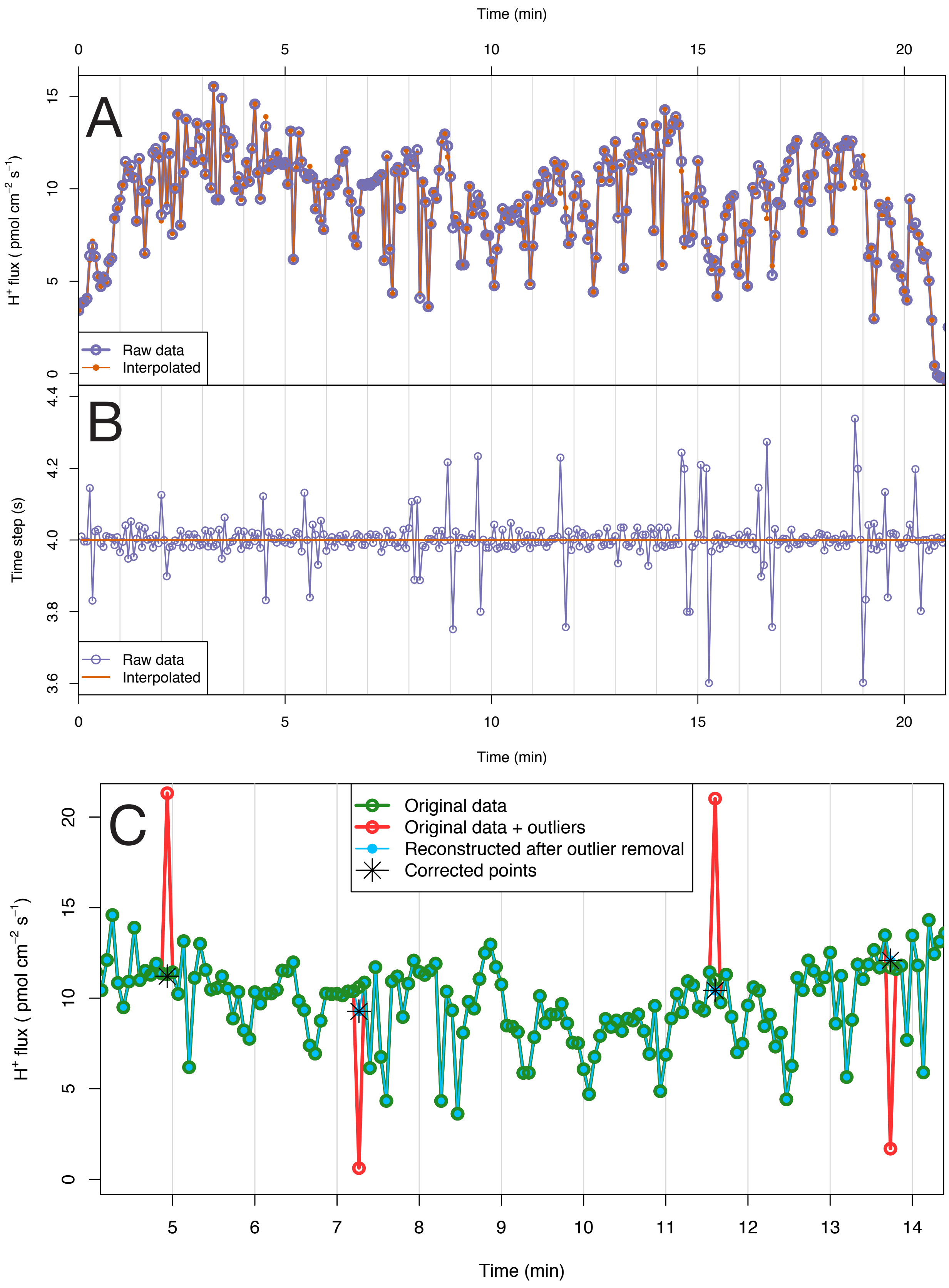

Fig. S5: Pre-filtering module showing interpolation (A-B) and outlier removal (C) in the illustrative H^+ flux series. A – Raw and series interpolated with loess. B – Time steps before and after interpolation. C – Removal of 4 artificial outliers inserted with 2 adding and 2

subtracting 10 pmol cm^{-2} s^{-1} to the original values, which have been successfully detected and restored to values laying close to the original data.

Tables

Table S1: Contingency tables comparing tip detection methods. Growth rate time series generated either by MetaMorph or the featured method were detrended and analyzed with a continuous wavelet transform, with significant periodic components being detected or not at each time point. Viewing each time point as a trial where each method can succeed or fail to detect oscillations allows testing the significance of the difference in the proportion of times each method succeeds with McNemar's paired proportion test.

	Featured method (unsmoothed)		
**Metamorph**	**Oscillation**	**No oscillation**	
**Oscillation**	93	0	$p = 5.9x10^{-20}$
**No oscillation**	87	134	

	Featured method (unsmoothed)		
**Featured method (smoothed)**	**Oscillation**	**No oscillation**	
**Oscillation**	180	79	$p = 3.4x10^{-18}$
**No oscillation**	0	55	

Miscellaneous files (Data and Videos)

Data S1: Code, data and supporting material. The folder is a clone from the current version of the pipeline available and maintained in the online repository GitHub (https://github.com/damineli/CHUKNORRIS). It contains the scripts in the programming language R in the folder 'src', featuring the 5 main analysis modules presented, the data analyzed here in corresponding folders under 'data' and a short usage tutorial.

Video S1: Pollen tube showing oscillatory growth with tip location trace obtained with MetaMorph and used to produce the single channel kymograph analyzed herein. The channel shown corresponds to YFP emission of the YC3.6 probe.

Video S2: Pollen tube showing cytosol retracting behavior that produces negative growth rate estimates with pronounced oscillations (Ca^{2+} spikes). The channel shown corresponds to YFP emission of the YC3.6 probe.
